# Meta-analysis of alcohol price and income elasticities – with corrections for publication bias

**DOI:** 10.1186/2191-1991-3-17

**Published:** 2013-07-24

**Authors:** Jon P Nelson

**Affiliations:** 1 Department of Economics, Pennsylvania State University, University Park, PA 16802, USA

**Keywords:** Alcohol demand, Price elasticity, Health production, Publication bias, Meta-analysis

## Abstract

**Background:**

This paper contributes to the evidence-base on prices and alcohol use by presenting meta-analytic summaries of price and income elasticities for alcohol beverages. The analysis improves on previous meta-analyses by correcting for outliers and publication bias.

**Methods:**

Adjusting for outliers is important to avoid assigning too much weight to studies with very small standard errors or large effect sizes. Trimmed samples are used for this purpose. Correcting for publication bias is important to avoid giving too much weight to studies that reflect selection by investigators or others involved with publication processes. Cumulative meta-analysis is proposed as a method to avoid or reduce publication bias, resulting in more robust estimates. The literature search obtained 182 primary studies for aggregate alcohol consumption, which exceeds the database used in previous reviews and meta-analyses.

**Results:**

For individual beverages, corrected price elasticities are smaller (less elastic) by 28-29 percent compared with consensus averages frequently used for alcohol beverages. The average price and income elasticities are: beer, -0.30 and 0.50; wine, -0.45 and 1.00; and spirits, -0.55 and 1.00. For total alcohol, the price elasticity is -0.50 and the income elasticity is 0.60.

**Conclusions:**

These new results imply that attempts to reduce alcohol consumption through price or tax increases will be less effective or more costly than previously claimed.

## Background

The objective of a meta-analytic review is quantitative synthesis of a body of literature. There are a number of obstacles that must be overcome for a synthesis to be successful. First, primary studies in economics usually employ a variety of data and statistical methods, which results in a dispersion of estimates due to methodological differences. For example, estimates from survey-based studies may be incompatible with traditional econometric studies using aggregate data. Second, factual heterogeneity may be present; it is not clear if sample estimates obtained with, say, older data sets are generated by the same population of consumers as more recent data series. Relatively simple meta-analytic methods are available for handling unobserved heterogeneity. Third, empirical estimates typically contain outliers that reflect sampling problems, estimation methods, or artifacts associated with data and methods. It is important to recognize that, in a meta-analysis, outliers can occur for both effect-sizes (elasticity estimates) and standard errors of the estimates. Fourth, the sample of estimates may contain publication selection bias, reflecting choices about “acceptable” estimates made by primary researchers, journal referees, and editors.^a^ Selection often means that reported estimates are biased toward larger, but less precise, values. Publication bias represents a threat to internal and external validity of all literature reviews, both traditional narrative reviews and quantitative syntheses.

This paper presents meta-analytic summaries of price and income elasticities for alcohol beverages, which correct for outliers and publication bias. The analysis builds on several recent meta-analyses, but those studies failed to give sufficient attention to these problems; see Fogarty [1], Gallet [2], and Wagenaar et al. [3]. The methods employed here are relatively straight-forward, but underutilized in the meta-analysis literature. First, for outliers, trimmed samples are employed, but trimming is along two dimensions: effect-sizes and size of primary standard errors. Due to weighting methods used in meta-analysis, especially the fixed-effect model, it is important that weighting is subjected to a sensitivity analysis. This can be accomplished by trimming samples for standard error outliers and by comparing fixed- and random-effect estimates. A 10% trimmed sample is used for this purpose. Second, publication bias is addressed using cumulative meta-analysis. Borenstein [4] initially proposed using cumulative meta-analysis, with ordering by precision, as a method for reducing publication bias; see also [5,6]. Briefly, primary data are ordered from high to low precision, and meta-analyses are run in a step-wise fashion using the first (most precise) estimate, then the first two estimates, and so on until the entire sample is fully analyzed. While other methods are available, advantages of this approach are that the analysis is transparent and focused on estimates with greater information value due to their greater precision. Less precise estimates are those that are more likely to be biased on selectivity grounds. This also is a form of sensitivity analysis since results from cumulative analyses can be compared to full sample estimates or summary estimates can be examined for shifts and drift as more studies are added to the analysis [4,7].

Previous meta-analyses provide a variety of summary averages for alcohol price and income elasticities, but for the most part these studies ignore publication bias. Gallet [2] presents unweighted medians by beverage and unweighted meta-regressions that focus on methodological dispersion. However, his meta-regressions pool all estimates, so marginal effects for various covariates are uniform across beverages. Gallet includes individual-level survey studies as well as aggregate studies in the analysis, but many survey studies report tax elasticities rather than price elasticities e.g., [8-10]. Price and tax elasticities are not comparable unless tax elasticities are transformed [[11], p. 72]. All tax elasticities are excluded here. Wagenaar et al. [3] report unweighted mean elasticities by beverage. Separate results are reported for aggregate and individual-level studies, but their analysis uses standardized effect sizes that are not interpretable as elasticities. While they demonstrate that increased prices have a negative effect on alcohol consumption, this leaves uncertain the magnitude of effects. Wagenaar et al. adjust for outliers by excluding a few estimates, but they fail to address publication bias. Fogarty [1] is more complete in several respects. He reports unweighted means and medians by beverage and provides average demand elasticities by country. Weighted meta-regressions are reported that focus on methodological differences among pooled primary estimates. However, prior to weighting, it is important to consider the influence of outliers. Fogarty [[1], p. 458] discusses publication bias, but concludes it is important for only beer price elasticities. Results reported below demonstrate a greater level of concern for this problem.

The present paper departs in several important ways compared to prior analyses of alcohol demand elasticities. First, the literature search uncovered a number of alcohol demand studies that were not included in prior analyses. Both published and unpublished studies are included. Inclusion/exclusion criteria for the search are designed to insure that primary studies are of comparable quality. Second, outliers are deleted using 10% trimmed samples, which is important if weighted means are to be used as summary statistics. Both fixed- and random-effect means are reported for full and trimmed samples. Third, publication bias is examined using funnel graphs and Egger’s intercept test, with standard errors corrected for clustering by country groups. Fourth, given the importance of publication bias in primary estimates, results from cumulative meta-analyses are used to correct summary statistics for this bias. Average price elasticities obtained in this paper are generally smaller (less elastic) compared to results in prior analyses and consensus averages. Several policy implications of this finding are discussed, including alcohol taxation and minimum pricing.

## Methods

### Database review

A search of the literature on alcohol demand was conducted during the months of August-October 2012. Recent meta-analyses provided a useful starting point through reference lists, but a number of additional studies have been added. Articles, chapters, books, reports, dissertations, and working papers were examined by the author for alcohol demand and alcohol-related outcomes, such as traffic fatalities, crime, labor productivity and wages, and youth consumption. Some econometric studies on alcohol harms include first-stage or structural demand estimates, which are easily overlooked. Search terms used were combinations of “alcohol” AND “demand” (OR “price” OR “elasticity” OR “tax” OR “cost”). Complementary searches also were conducted using “beer,” “wine,” “liquor,” and “distilled spirits.” Databases searched were AgEcon Search, EconLit, JSTOR, RePEc, SSRN, and on-line retrieval engines for EBSCO Host, ProQuest, ScienceDirect Journals, and Wiley Online Library. Numerous Google searches also were conducted using similar search terms and keywords, which were helpful in retrieving the “grey literature” such as working papers and other unpublished materials.^b^ References in the studies were used for ancestral-based retrievals. The search was restricted to materials in the English language, but not limited to articles in peer-reviewed journals. Table 1 summarizes search and selection processes, where a total of 578 primary studies were recovered and examined as shown in additional file 1. Alcohol studies bibliography. A hard copy was obtained for each of these studies in its entirety. Abstracts, tables, and other summaries were screened by the author to select alcohol demand studies that provide comparable estimates for price and income elasticities of alcohol beverages. There are 182 studies that provide elasticity estimates for beer, wine, distilled spirits, or total alcohol.

**Table 1:** Summary of database review – 578 alcohol studies

**Review step**	**Results of data extraction**
1.	***Total alcohol-related studies retrieved in search: 578 studies***
	a. Excluded individual-level studies and alcohol-harms studies: 223 studies
2.	***Demand studies examined for inclusion in meta-analysis: 355 studies***
	a. Excluded studies in Fogarty [4] or Gallet [5] or Wagenaar et al. [6]: 58. Reasons: taxation study–12; older data (pre-1950)–9; std. errors missing–8; prices missing /income only–9 firm/brand/varietal/survey data–10;duplicate study–3; and linear model or other reason–7.
	b. Excluded aggregate studies in Wagenaar et al. [6]: 14. Reasons: taxation study–5; no. std. errors or no elasticities–5; and duplicate, older data or other–4.
	c. Excluded individual-level studies in Wagenaar et al. [6]: 33
3.	***Remaining studies examined: 297 studies***
	a. Additional excluded studies: 115. Reasons: taxation study–5; std.errors missing–7; prices missing/income only–27; duplicate study–5; firm/brand/varietal/survey data–28; no elasticities reported–2; and linear model or poor data--22.
4.	***Total studies containing aggregate elasticities: 182 (any alcohol beverage or total alcohol)***
	a. Included studies not reported in Fogarty [4] or Gallet [5]: 64
	b. Included studies not reported in Wagenaar et al. [6]: 135
	c. Type of publication for 182 studies: articles–127; chapters & books–17; reports–4; dissertations–8; working papers and reports–26
5*.*	***Total primary studies analyzed by beverage***
	a. Comparable studies for beer elasticities: 112 (93 published, 19 unpublished)
	b. Comparable studies for wine elasticities: 104 (89 published, 15 unpublished)
	c. Comparable studies for distilled spirits elasticities: 111 (97 published, 14 unpublished)
	d. Comparable studies for total alcohol elasticities: 66 (55 published, 11 unpublished)

A complete bibliography listing of the 578 studies and tables with the extracted data for this study are available as Additional file 1 and Additional file 2. Comparisons with prior studies are based on the supplemental tables from [2,3] and published references in [1].

### Extraction of estimates

The present analysis is based on estimates obtained from 112 primary studies for beer elasticities, 104 studies for wine, 111 studies for distilled spirits, and 66 studies for total alcohol demand. Among the 182 studies, there are 64 studies that were not included in Fogarty [1] or Gallet [2], and 58 studies included in their analyses that are excluded here (reasons are detailed in Table 1). Compared to Wagenaar et al. [3], there are 47 studies excluded (mostly individual-level survey studies) and 135 studies that were not included in their analysis. Data used in the present analysis reflect the following inclusion/exclusion criteria:

•Aggregate studies are included that provide price and income elasticities for aggregate quantity consumed, and a reported standard error (t-statistic, p-value, confidence interval) for the elasticity or for price and income coefficients. Household survey studies are included if they provide population-level elasticities and standard errors.

•Aggregate studies are excluded that use alcohol taxes as proxies for prices. Aggregate studies and household surveys are excluded if prices are not included. Studies are excluded if based predominantly on older data (pre-1950). Duplicates are excluded.

•Individual-level survey studies are excluded because alcohol consumption is self-reported, prices are imputed or taxes are used as price proxies, and “elasticities” cover a wide range of drinking behaviors (participation, prevalence, frequency, binging, etc.). These elasticities are not comparable to aggregate quantity elasticities.

•Studies are excluded that use firm-level data, brand data, and narrowly-defined beverage categories, such as vodka and wine varietals.

•About 18% of included studies were found in the grey literature (working papers, reports, dissertations) compared to, say, only 3% in Wagenaar et al. [3].

Data from each study were collected and recorded by the author, including page or table numbers for primary estimates. No elasticity values were estimated, which is a reason for excluding most linear model estimates. Empirical studies in economics frequently report multiple estimates based on the same data or major portions of the same data set. Using all estimates creates intra-study dependence or correlation, which will bias summary statistics. More weight is assigned to primary studies with multiple estimates than to studies with one or two estimates. Equally important, using multiple estimates will bias standard errors of summary statistics, with understatement of errors being the generally expected outcome [[7], p. 226]. There are several possible solutions to dependence problems, including intra-study averages, hierarchical/multi-level models, and cluster robust standard errors [12]. Inter-study dependence also occurs when primary investigators use the same data to estimate different models in separate publications or different investigators use the same data or very similar data. Following Fogarty [[1], p. 437], collection of effect sizes was restricted to one or two estimates per primary study or model. In general, the primary author’s preferred result was selected from each study and an important variation was used to capture different model specifications or estimation methods. While this restriction reduces intra-study dependence, it does not address inter-study dependence. In particular, several authors have published multiple studies using similar data sets, such as country-level models using annual time series. These authors also tend to use different econometric methods in each study, resulting in different “treatment effects.” Meta-regressions reported below include cluster robust standard errors for seven different country groupings, which address potential data dependencies at this aggregate level. The extracted data are in Additional file 2.

## Results

### Results for summary averages

Weighted and unweighted averages are reported in Table 2, with standard errors also reported for weighted means. Summaries of averages in three prior analyses are reported for comparison. Unweighted medians are reported for full samples used in this study. Trimmed samples are obtained by eliminating 10% of each sample by discarding 2.5% of the largest effect-sizes; 2.5% of the smallest effect-sizes; 2.5% of the largest standard errors; and 2.5% of the smallest standard errors. Trimming the sample has no effect on unweighted medians and only a small effect on unweighted means. However, standard errors for unweighted means are reduced by about 25-35%. Weighted means for trimmed samples are reported using fixed-effect (FE) and random-effects (RE) models. In meta-analysis, fixed-effect models use inverse variances for weights, with dispersion in estimates due solely to stochastic sampling error in each primary study [13,14]. That is, all studies are viewed as estimating a *common*, or fixed, population effect size. Estimates with smaller standard errors provide more precise information about the population value and are given greater weight. The *plural* or random-effects model also accounts for variation in population values by estimating a common inter-study variance based on observed dispersion of estimates, which is added to the study-level variance. Inverses of combined variances provide weights for each effect size. Thus, in the random-effects model, true effect sizes are similar or comparable, but not identical across studies. This is the simplest procedure for addressing heterogeneity among estimates. Compared to fixed-effects, random-effect models assign greater weight to less precise studies and, as pointed out by Fogarty [1], p. 455], random-effect weights also reduce the importance of outliers among standard errors.^c^

**Table 2:** Average price and income elasticities for alcohol beverages

**Study/sample (no. obs. price, income)**	**Price elasticity (se)**	**Income elasticity (se)**
**Fogarty**[1]**– unwt. medians**		
beer (154, 121)	−0.33	0.67
wine (155, 123)	−0.55	1.00
spirits (162, 123)	−0.76	1.24
alcohol	na	na
**Gallet**[2]**– unwt. means**		
beer (315, 278)	−0.36	0.39
wine (300, 240)	−0.70	1.10
spirits (294, 245)	−0.68	1.00
alcohol (263, 250)	−0.50	0.50
**Wagenaar et al.**[3]**– unwt. means**		
beer (105)	−0.46	na
wine (93)	−0.69	na
spirits (103)	−0.80	na
alcohol (91)	−0.51	na
**Full samples – unwt. medians**		
beer (191, 169)	−0.32	0.66
wine (197, 172)	−0.57	1.06
spirits (202, 179)	−0.67	1.24
alcohol (117, 88)	−0.54	0.70
**Trimmed samples – FE means**		
beer (172, 151)	−0.26 (.01)	0.49 (.01)
wine (178, 155)	−0.34 (.01)	0.64 (.01)
spirits (182, 160)	−0.49 (.01)	0.87 (.01)
alcohol (105, 77)	−0.46 (.01)	0.48 (.01)
**Trimmed samples – RE means**		
beer (172, 151)	−0.35 (.02)	0.58 (.03)
wine (178, 155)	−0.58 (.03)	1.12 (.06)
spirits (182, 160)	−0.64 (.03)	1.15 (.04)
alcohol (105, 77)	−0.58 (.03)	0.66 (.04)

Standard errors (se) in parentheses, with all p-values < 0.001; FE is fixed-effect and RE is random-effects weighted means, obtained using CMA 2.0 [15].

Unweighted averages are similar for the several analyses, suggesting that prior analyses are not biased on sampling grounds. Average beer price elasticities range from -0.32 to -0.46, with the largest average reported in [3]; wine prices, -0.55 to -0.70, with the largest reported in [2]; spirits prices, -0.67 to -0.80, with the largest reported in [3]; and total alcohol prices, -0.50 to -0.54. For income, the ranges are beer, 0.39 to 0.67; wine, 1.00 to 1.10; spirits, 1.00 to 1.24; and total alcohol, 0.50 to 0.70. Combing results, “consensus” averages for price elasticities are approximately: beer price, -0.40; wine price, -0.65; spirits price, -0.75; and total alcohol price, -0.50. Unweighted averages for income elasticities are approximately: beer, 0.60; wine, 1.00; spirits, 1.20; and total alcohol, 0.60.

Results for weighted means indicate less elastic demands, especially FE means for prices. The FE and RE means for trimmed-samples are: beer price, -0.26 and -0.35; wine price, -0.34 and -0.58; spirits price, -0.49 and -0.64; and total alcohol price, -0.46 and -0.58. For income, FE and RE means are: beer, 0.49 and 0.58; wine, 0.64 and 1.12; spirits, 0.87 and 1.15; and total alcohol, 0.48 and 0.66. Hence, using the RE model increases price and income elasticities. The results illustrate the importance of different weighting schemes. Because random-effects account for the second-level variance at study levels, it is the preferred approach when unobserved heterogeneity is known to be present. However, if publication bias is detected, the estimates need to be refined further. As pointed out by Borenstein et al. [[7], p. 378], “. . . if these studies are a biased sample of all possible studies, then the mean effect reported by a meta-analysis will reflect this bias”.

### Results for publication bias

This section reports tests for publication bias using two techniques. First, funnel graphs are presented for price elasticities for beer, wine, and spirits. Graphs for total alcohol and income elasticities are omitted in the interest of space, but can be obtained by request. Second, results for Egger-intercept tests are reported using meta-regressions. Funnel plots and meta-regressions indicate moderate to severe publication bias for all samples and estimates, except for beer income elasticities.

**Figure 1:**
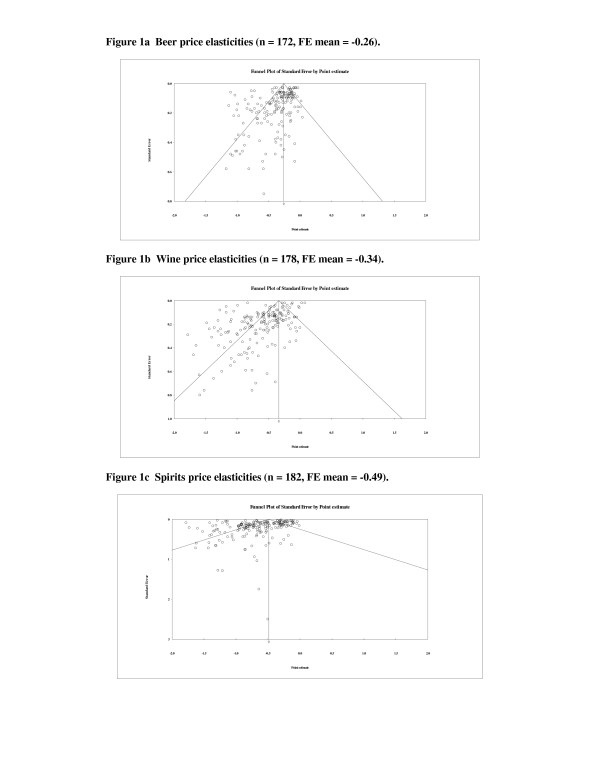
a Beer price elasticities (n = 172, FE mean = -0.26). b Wine price elasticities (n = 178, FE mean = -0.34). c Spirits price elasticities (n = 182, FE mean = -0.49).

A standard method for detecting publication bias is a funnel graph. Briefly, the horizontal scale measures the effect size and the vertical scale measures the standard error (or precision). In the absence of publication bias, a plot of effects against their errors should be symmetric about a weighted mean. However, a “funnel-shaped” plot is expected due to heteroskedasticity as larger effects tend on average to have larger errors regardless of publication bias. In the presence of bias, a plot can be either less dense or asymmetric. First, suppose the true effect in the population is zero. If studies with significant results are more likely to be published, bias is revealed in the plot as an empty area around zero that results from non-publication of insignificant or null results regardless of direction or sign. Second, suppose instead that the true effect is not zero, but rather some small or moderate value. Bias will appear as an asymmetric plot as studies with small effects and large errors are less likely to be published or reported. Asymmetry also reveals the direction of bias as successful publication tends to be associated with both sign and significance of results. Funnel plots are an informal method, so it is important to recognize that a skewed plot also can result from underlying heterogeneity in the sample. Figures 1a – 1c show funnel graphs for price elasticities for beer, wine, and spirits. The vertical line in each graph is the fixed-effect mean, reflecting a concern that random-effects are partly due to publication bias [[16], p. 82]. Diagonal lines indicate 95% confidence intervals. The graphs indicate asymmetry with a negative bias in primary estimates, which implies that reported estimates tend toward more elastic values.

A second approach to detecting and measuring publication bias is to estimate a meta-regression with standard errors or precision as the sole covariate. In the absence of publication bias, effect size estimates and their standard errors are independent. If not, they are correlated, and summary statistics and standard errors are biased. For the *i*-th observation (*i = 1, . . . , N*), a regression-based test of this association can be represented as [16-19].

(1) $$ES_{i}\;=\;\beta_{1}\;+\;\beta_{0}Se_{i}\;+\;\epsilon_{i}$$

where *ES* is the estimated price elasticity in study *i*, *Se* is its standard error, and *ϵ* is a stochastic error term. In the absence of selection, observed effects should vary randomly about the true effect size, *β*_1_, independent of standard errors. However, if model specifications are selected based on significance of the main covariates, effect sizes will vary directly with standard errors *Se*. Because estimates are inherently heteroskedastic, it is appropriate to divide Equation (1) by the standard error to yield [20,21].

(2) $$t_{i}\;=\;\beta_{0}\;+\;\beta_{1}\left(1/Se_{i}\right)\;+\;\upsilon_{i}$$

where *t*_*i*_ is the *t*-statistic for the *i*-th observation, 1*/Se*_*i*_ is its precision, and *υ*_*i*_ is an error term corrected for heteroskedasticity. Estimating Equation (2) by OLS is equivalent to estimating Equation (1) by weighted least-squares with inverse variance weights. In Equation (2), a test of H_0_: *β*_*1*_ = 0 is a test of absence of publication bias or the funnel-asymmetry test (FAT). If FAT rejects symmetry, the estimate of *β*_*0*_ is interpreted as the effect size corrected for bias, referred to as the precision-effect test (PET); see [16].

Table 3 shows results for Egger intercept or FAT-PET regressions for price and income elasticities, estimated using trimmed samples. Applying FAT, all intercepts are statistically significant with exception of the intercept for beer income elasticities. Absolute intercept values are between 1.0 and 2.0, which is consistent with substantial selection bias [22]. Applying PET, precision coefficients indicate the following average price elasticities: beer, -0.17; wine, -0.23; spirits, -0.39; and total alcohol, -0.32. These are relatively inelastic values, indicating that bias is present in unweighted or uncorrected averages. Income elasticities are: beer, 0.40; wine, 0.39; spirits, 0.66; and total alcohol, 0.32. Robust standard errors are obtained by clustering on seven country-level groups, reflecting a concern that there is correlation among errors due to similar data, similar alcohol-institutions (e.g., state monopolies in Nordic countries), and similar drinking cultures. Overall, results from funnel plots and regression-based tests indicate moderate to substantial publication bias, which is reflected in larger, more elastic values, especially for price elasticities.

**Table 3:** Results for regression tests for publication bias

**Alcohol beverage**	**Intercept (se)**	**Precision (se)**
**Beer prices**		
(n = 172, R^2^ = 0.238)	−1.425 (.217)*	−0.172 (.045)*
**Beer income**		
(n = 151, R^2^ = 0.272)	1.266 (1.20)	0.405 (.183)*
**Wine prices**		
(n = 178, R^2^ = 0.230)	−2.057 (.509)*	−0.228 (.037)*
**Wine income**		
(n = 155, R^2^ = 0.176)	3.113 (.852)*	0.388 (.183)*
**Spirits prices**		
(n = 182, R^2^ = 0.353)	−1.530 (.450)*	−0.388 (.083)*
**Spirits income**		
(n = 160, R^2^ = 0.459)	2.299 (.640)*	0.657 (.141)*
**Total alcohol prices**		
(n = 105, R^2^ = 0.284)	−1.830 (.917)*	−0.316 (.135)*
**Total alcohol income**		
(n = 77, R^2^ = 0.419)	2.113 (.327)*	0.322 (.068)*

Asterisks indicate the robust t-statistic is greater than 2.0 in absolute value. Robust standard errors (se) in parentheses obtained using cluster options in Stata 12, adjusted for seven country groups. Country-level groups are: (1) Australia and New Zealand; (2) Canada; (3) Europe (Belgium, Cyprus, Czech Republic, France, Germany, Greece, Hungary, Italy, Netherlands, Poland, Portugal, Romania, Spain, Multiple); (4) Nordic (Denmark, Finland, Norway, Sweden); (5) United Kingdom and Ireland; (6) United States; and (7) Other (Chile, China, India, Japan, Kenya, Russia, Taiwan, Tanzania, Turkey).

### Results for cumulative meta-analyses

Once there is evidence of bias, there are several possible ways it might be dealt with as a statistical problem [18,23]. Although bias is probably present in most literatures, its extent can be modest [22,24]. Correcting for bias helps ensure that resulting summary statistics are robust. Borenstein [[4], p. 198] proposed cumulative meta-analysis as a correction method:

For this purpose the studies would be sorted in the sequence from largest to smallest (or of most precise to least precise), and a cumulative meta-analysis preformed with the addition of each study. If the point estimate has stabilized with the inclusion of the larger studies and does not shift with the addition of smaller studies, then there is no reason to assume that the inclusion of smaller [or less precise] studies has injected a bias (i.e., since it is the smaller studies in which study selection is likely to be greatest).

Borenstein’s proposal leaves uncertain the meaning of “stabilized” or magnitude of a “shift,” which might vary by analyst. A fixed or prior standard for cumulative averages makes for a transparent correction, although any method used should also allow for a sensitivity analysis. The analysis here uses the 50th percentile as a cutoff (median cumulative average). For a sensitivity analysis, results also are reported for 25th and 75th percentiles. Reflecting a concern with heterogeneity among estimates, summary averages use random-effects models. Hence, the cumulative weighted averages reported here are corrected for outliers, dependence, heterogeneity, and publication bias.

Table 4 displays results for cumulative analyses, with means at the 50th percentiles reported in top-most rows. Compared to “consensus” averages, the beer price elasticity is about 28% smaller in absolute value (-0.29 compared to -0.40); wine price is 29% smaller (-0.46 compared to -0.65); and spirits price is 28% smaller (-0.54 compared to -0.75). These errors are smaller than estimates from similar corrections [25], but they are still substantial and robust across beverages. Total alcohol price elasticity is virtually unchanged (-0.48 compared to -0.50). This last result provides support for a conjecture by Clements [26] that price elasticities for broadly defined commodities are about minus one-half. Income elasticities for wine and total alcohol are unchanged, but the beer income elasticity is reduced by 15% (0.50 compared to 0.60) and spirits income elasticity is 20% smaller (1.00 compared to 1.20). For a sensitivity analysis, other results indicate that less precise estimates cause some shift, although it is fairly small. Compared to unweighted averages obtained with full samples in Table 2, averages at the 50th percentile are smaller (less elastic) for prices by about 15-20% and smaller for income by 10-20%.

**Table 4:** Cumulative meta-analysis for alcohol price and income elasticities

**Study/sample**	**Price elasticity (se)**	**Income elasticity (se)**
**Cumulative analysis**: 50th percentile (no. obs. for price, income)		
beer (86, 76)	−0.29 (.02)	0.51 (.05)
wine (89, 78)	−0.46 (.04)	0.99 (.08)
spirits (91, 80)	−0.54 (.04)	1.00 (.06)
alcohol (52, 38)	−0.49 (.05)	0.59 (.05)
**Additional results**: 25th percentile (no. obs. for price, income)		
beer (43, 38)	−0.26 (.03)	0.51 (.07)
wine (44, 39)	−0.43 (.05)	0.83 (.11)
spirits (46, 40)	−0.52 (.06)	0.83 (.07)
alcohol (26, 19)	−0.46 (.08)	0.54 (.06)
**Additional results**: 75th percentile (no. obs. for price, income)		
beer (129, 113)	−0.33 (.02)	0.55 (.04)
wine (134, 116)	−0.52 (.03)	1.09 (.07)
spirits (136, 120)	−0.61 (.03)	1.14 (.05)
alcohol (79, 58)	−0.51 (.04)	0.63 (.04)

Data ordered from most precise (smallest std error) to least precise. Trimmed samples are used. Random-effects means with standard errors (se) in parentheses, all p-values < 0.001. Results obtained using CMA 2.0 [15].

In summary, the beer price elasticity is estimated to be -0.29 (95% confidence interval -0.25 to -0.33); wine price, -0.46 (-0.38 to -0.54); spirits price, -0.54 (-0.46 to -0.62); and alcohol price, -0.49 (-0.39 to -0.59). The beer income elasticity is 0.51 (95% confidence interval 0.41 to 0.61); wine income, 0.99 (0.83 to 1.15); spirits income, 1.00 (0.88 to 1.12); and alcohol income, 0.59 (0.49 to 0.69).

### Have price and income elasticities changed over time?

Price and income elasticities vary across studies for many possible systematic reasons, including primary study methodology, country of origin, publication date, sample time period, and so forth. Fogarty [1] and Gallet [2] report meta-regression results for numerous potential moderators, especially methodological covariates. However, in both cases, the meta-regressions combine elasticities across beverages, leaving results at the beverage level uncertain. As demonstrated above, there are noticeable differences across beverages for price and income elasticities, especially for beer. One interesting relationship explored by Fogarty [1] is the possibility of a time trend in elasticity estimates. Using average dates from primary study data, he projects that alcohol beverages are modestly more price elastic after 1953 and less income elastic after 1965. Although precise explanations for these results are not given, it is reasonable to expect that changes in consumer attitudes toward alcohol might play some role. In any event, additional tests of the magnitude of change are in order.

A cumulative meta-analysis also can be used to explore possible time trends, with a ranking of estimates according to the mid-point of each primary study’s data. I ranked the estimates from most recent (2005) to oldest (1949), and performed the analysis in reverse chronological order with older estimates progressively added to the sample. Table 5 displays results for each beverage for price and income elasticities for two time periods, 1975-2005 and 1949-2005. Over time, there are modest increases in price elasticities and modest declines in income elasticities. As a check on these results, univariate mixed-effect regressions were estimated, with a covariate for the average primary sample date. Slope coefficients for beer, spirits and alcohol prices were small and negative, but never statistically significant. The slope coefficient for wine prices was positive, but not significant. For income elasticities, beer and wine slope coefficients were statistically insignificant. For spirits and alcohol, slope coefficients were negative and significant. Hence, the averages in Table 5 and regression results suggest that price elasticities are stable over time. Spirits and alcohol income elasticities are smaller over time, but, on average, any changes are modest.

**Table 5:** Cumulative meta-analysis by primary sample time period

**Beverage (no. obs., price, income)**	**Price elasticity (se)**	**Income elasticity (se)**
**Beer**		
1975-2004 (98, 84)	−0.38 (.03)	0.63 (.05)
1949-2004 (172, 151 )	−0.35 (.02)	0.58 (.03)
**Wine**		
1975-2004 (108, 89)	−0.57 (.04)	1.10 (.07)
1954-2004 (178, 155)	−0.58 (.03)	1.12 (.06)
**Spirits**		
1975-2004 (100, 86)	−0.66 (.04)	1.09 (.06)
1954-2004 (182, 160)	−0.64 (.03)	1.15 (.04)
**Alcohol**		
1975-2005 (76, 60)	−0.60 (.04)	0.61 (.04)
1960-2005 (105, 77)	−0.58 (.03)	0.66 (.04)

Data ordered from most recent primary sample date (mid-point) to oldest. Trimmed samples are used. Random-effects means with standard errors (se) in parentheses, all p-values < 0.001. Results obtained using CMA 2.0 [15].

## Discussion

Results in this paper for price elasticities have important implications for alcohol policy debates. Interest in price elasticities by policymakers has heightened in recent years as attention has focused on reducing average population-level consumption as part of a policy for addressing problem drinking and abusive alcohol consumption [11,27,28]. In part, this concern is motivated by a lognormal single-peaked (unimodal) distribution model or Ledermann hypothesis for alcohol consumption. This hypothesis holds that a society’s alcohol problems are directly-related to average levels of consumption, even if only a small percentage of the population is categorized as “problem drinkers” [29]. The relationship has been interpreted to indicate that average consumption causally determines heavy drinking prevalence, possibly through a contagious social environment or “drinking culture.” This view is expressed in the continuing interest in alcohol taxes [11], recent interest in minimum pricing of alcohol [30,31], and concern with the increased “affordability” of alcohol beverages over time or across countries [32,33]. However, as explained by Duffy [34], heavy drinkers account for a large proportion of total alcohol consumption, so a statistical relationship between average consumption in a country and the percentage of heavy drinkers should be expected, and does not necessarily imply a causal relationship.

## Conclusions

The average price elasticities reported here imply that reducing alcohol consumption through price or tax increases will be less effective or more costly than previously suggested or claimed [35-38]. Cumulative average price elasticities are 28-29% smaller than “consensus” estimates, with a price inelastic demand for all three beverages (-0.30 to -0.55) and total alcohol (-0.50). On the other hand, income elasticities are more substantial for all beverages. Affordability of alcohol is therefore likely to be significantly influenced by rising real incomes. Further, recent policy proposals, such as minimum pricing, fail to account for differences in elasticities according to drinking levels or patterns. Even if the distribution of consumption is lognormal, it does not follow that responses to prices are uniform across subpopulations of drinkers. Available evidence from survey studies indicates that demands by heavy drinkers are less responsive to price compared to the general population [39,40]. While many moderate drinkers probably respond to changes in prices of most alcohol beverages, it does not follow that an across-the-board price or taxation policy will directly reduce heavy drinking. In sum, demand heterogeneity exists for alcohol beverages and alcohol consumers.

## Endnotes

^a^Card and Kruger [17] attribute publication bias to three sources: (1) reviewers and journal editors may be predisposed to accept papers that support conventional views such as large negative price elasticities; (2) reviewers and journals tend to favor papers with statistically significant results; and (3) primary researchers use t-statistics of two or more for the main covariates as a guide for model specification and selection. See also [41,42].

^b^The grey literature search was aided by files maintained by the author since 1990. A summary is: unpublished working papers and dissertations/reports with dates prior to year 2000 (11 items); and unpublished working papers and dissertations/reports with dates from 2000-2012 (22 items). All unpublished materials are listed in the Additional file 1.

^c^Consider three price elasticity estimates and their standard errors: -0.10 (.01); -0.50 (.20); and -1.20 (.60). The median is -0.50 and the unweighted mean is -0.90. The FE mean uses inverse variances for weights, so the first estimate receives a weight proportional to 1/(.01)^2^ = 10,000, while the weights for the other two estimates are proportional to 25 and 2.8. The FE mean for this sample is -0.10 (.01) and the random-effects mean is -0.37 (.22). The example illustrates the importance of outliers for standard errors in meta-analysis, especially FE models.

## Abbreviations

FE: Fixed-effect; RE: Random-effects; FAT: Funnel-asymmetry test; PET: Precision-effect test.

## Competing interests

Research leading to this paper was supported in part by the International Center for Alcohol Policies, Washington, DC. This paper presents the work product, findings, viewpoints, and conclusions solely of the author. The views expressed are not necessarily those of ICAP or any of ICAP’s sponsoring companies.

## Supplementary Material

Additional file 1 Alcohol studies bibliography. Click here for file

Additional file 2: Table S1 Beer price and income elasticities. Table S2. Wine price and income elasticities. Table S3. Spirits price and income elasticities. Table S4. Alcohol price and income elasticities. Click here for file
