# Supplementary material for: Meta-analysis of alcohol price and income elasticities – with corrections for publication bias
Source: Health Econ Rev. 2013 Jul 24;3:17. doi: 10.1186/2191-1991-3-17 (PMC3722038; doi:10.1186/2191-1991-3-17)
Supplement: Additional file 1 — Alcohol studies bibliography. [file 2191-1991-3-17-S1.docx]

**SUPPLEMENTAL TABLES:**

**Supplemental Table S1**. Beer Price and Income Elasticities

**Supplemental Table S2**. Wine Price and Income Elasticities

**Supplemental Table S3**. Spirits Price and Income Elasticities

**Supplemental Table S4**. Alcohol Price and Income Elasticities

**May 1, 2013**

**Supplemental Table S1**. Beer Price and Income Elasticities

| **Study (year published)** | **Country** | **Beer price (se)** | **Beer income (se)** |
| --- | --- | --- | --- |
| Adrian & Ferguson (1987) | Canada | -037 (.15), -0.84 (.17) | 0.23 (.12), 1.54 (.43) |
| Alley et al. (1992) | Canada, BC | -0.15 (.26) | 0.06 (.02) |
| Andrikopoulos et al. (1997) | Canada, ON | -0.48 (.13), -1.02 (.46) | 0.96 (.17) |
| Andrikopoulos et al. (1997) | Canada, ON | -0.08 (.07), -1.00 (.46) |  |
| Andrikoupolos & Loizides (2000) | Cyprus | -0.35 (.33), -1.00 (.18) | 1.30 (.29), 1.02 (.11) |
| Angulo et al. (2001) | Spain | -1.17 (.58) | 0.87 (.44) |
| Ashton & Casswell (1987) | New Zealand | -0.45 (.28) | 0.21 (.18) |
| Barnes (1984) | Canada | -0.99 (.14) | -0.10 (.03) |
| Bentzen et al. (1997) | Denmark | -0.47 (.14) | 0.50 (.14) |
| Bentzen et al. (1997) | Norway | -0.39 (.15) | 0.43 (.14) |
| Bentzen et al. (1997) | Sweden | -0.67 (.27) | 1.44 (.44) |
| Berggren (1997) | Sweden | -0.32 (.06) | 0.63 (.04) |
| Blake & Nied (1997) | UK | -0.95 (.48), -1.27 (.64) | 0.83 (.42), 0.81 (.40) |
| Chang et al. (2002) | Australia | -0.82 (.14) | 1.04 (.22) |
| Chetty (2009) | USA | -0.88 (.42) | 0.23 (.10) |
| Clements & Johnson (1983) | Australia | -0.09 (.03), -0.36 (.07) | 0.83 (.06), 0.80 (.15) |
| Clements & Selvanathan (1988) | UK | -0.21 (.10) | 0.41 (.08) |
| Clements & Selvanathan (1988) | USA | -0.09 (.08) | 0.76 (.11) |
| Clements & Selvanathan (1991) | Australia | -0.43 (.06), -0.15 (.02) | 0.73 (.05), 0.73 (.05) |
| Clements et al. (1997) | Australia | -0.40 (.03) | 0.81 (.14) |
| Clements et al. (1997) | Canada | -0.31(.07) | 0.74 (1.15) |
| Clements et al. (1997) | Finland | -0.61 (.15) | 0.45 (.08) |
| Clements et al. (1997) | New Zealand | -0.37 (.08) | 0.84 (.39) |
| Clements et al. (1997) | Sweden | -0.30 (.38) | 0.21 (.07) |
| Clements et al. (1997) | UK | -0.44 (.04) | 0.82 (.13) |
| Clements & Daryl (2005) | Australia | -0.17 (.09) |  |
| Collis et al. (2010) | UK | -0.77 (.05), -1.10 (.06) | 0.31 (.12), 0.22 (.09) |
| Comanor & Wilson (1974) | USA | -1.39 (.60), -0.56 (.24) | -0.46 (.29), -0.18 (.11) |
| Crawford & Tanner (1995) | UK | -0.67 (.24) |  |
| Crawford et al. (1999) | UK | -0.76 (.09) |  |
| Crooks (1989) | UK | -1.05 (.22) | 0.92 (.07) |
| Duffy (1982a) | UK | +0.04 (.23) | 0.79 (.13) |
| Duffy (1982b) | UK | -0.14 (.20), -0.17 (.36) | 0.66 (.23), 0.49 (.24) |
| Duffy (1983) | UK | +0.20 (.17), +0.23 (.15) | 0.85 (.19), 1.07 (.09) |
| Duffy (1987) | UK | -0.29 (.10), -0.36 (.12) | 0.60 (.08), 0.71 (.15) |
| Duffy (1990) | UK | -0.48 (.14), -0.27 (.09) | 0.73 (.16), 0.65 (.13) |
| Duffy (1991) | UK | -0.09 (.10) | 0.54 (.12) |
| Duffy (1995) | UK | -0.29 (.20), -0.03 (.16) | 1.44 (.28), 0.88 (.28) |
| Duffy (2001) | UK | -0.12 (.06), -0.13 (.08) | 0.79 (.04), 0.76 (.05) |
| Duffy (2002) | UK | -0.37 (.98), -0.39 (.48) | 0.98 (1.02), 0.93 (1.88) |
| Duffy (2003) | UK | -0.40 (.19) | 0.98 (.20) |
| Eakins & Gallagher (2003) | Ireland | -0.53 (.13), -0.76 (.19) | 0.16 (.03), 1.03 (.22) |
| Fang (2003) | China | -0.59 (.53), -0.90 (.81) | 0.85 (.36) |
| Gallet (2007) | USA | -0.03 (.03) | 0.16 (.06) |
| Gallet & List (1998) | USA | -1.72 (.31) | -0.26 (.21) |
| Gao et al. (1995) | USA | -0.23 (.13) | -0.09 (.05) |
| Goldschmidt (1990) | Australia | -0.10 (.03) | 0.73 (.13) |
| Gruber et al. (2002) | Canada | -0.19 (.09) |  |
| Gruenewald et al. (2006) | Sweden | -1.70 (.18) | 0.19 (.39) |
| Hagan & Waterson (1983) | UK | -0.34 (.17) | 0.04 (.34) |
| Heien & Pompelli (1989) | USA | -0.84 (.95) | 1.94 (.10) |
| Hogarty & Elzinga (1972) | USA | -0.89 (.35) | 0.43 (.17) |
| Holm (1995) | Finland | -0.51 (.26) | 1.47 (.74) |
| Holm & Suoniemi (1992) | Finland | -0.30 (.42) |  |
| Huang (2003) | UK | -1.03 (.08) | 0.55 (.08) |
| Janda et al. (2010) | Czech Republic | -0.97 (.22) | 1.33 (.03) |
| Jithitkulchai (2010) | USA | -0.57 (.75) | 0.02 (.02) |
| Johnson & Oksanen (1974) | Canada | -0.22 (.07), -0.38 (.13) | 0.04 (.05), 0.06 (.09) |
| Johnson & Oksanen (1977) | Canada | -0.25 (.08), -0.29 (.09) | -0.01 (.04), -0.10 (.36) |
| Johnson et al. (1992) | Canada | -0.26 (.06), -0.14 (.05) | 0.38 (.17), 0.27 (.10) |
| Johnson LW (1985) | UK | -0.42 (.10) | 0.56 (.07) |
| Jones (1989) | UK | -0.27 (.13), -0.40 (.12) | 0.31 (.18), 0.50 (.16) |
| Keane (1965) | USA | -0.69 (.20) | 0.27 (.10) |
| Lariviere et al. (2000) | Canada, ON | -0.35 (.37), -0.38 (.40) |  |
| Lau (1975) | Canada | -0.08 (.41) | 0.40 (.23) |
| Lee & Tremblay (1992) | USA | -0.61 (.27), -0.81 (.36) | 0.08 (.09), 0.11 (.13) |
| Leong & Wang (1994) | USA, FL | -0.52 (.19), -0.98 (.35) | 1.00 (.94) |
| Mangeloja & Pehkonen (2009) | Finland | -0.21 (.21) | -0.28 (.28) |
| McGuinness (1983) | UK | -0.30 (.09) | 0.13 (.15) |
| Meyerhoefer et al. (2005) | Romania | -1.28 (.10) | 0.84 (.04) |
| Nelson (1990a) | USA | -0.56 (.20) | 0.48 (.20) |
| Nelson (1990b) | USA | -0.56 (.17) | 0.38 (.17) |
| Nelson (1997) | USA | -0.27 (.11), -0.16 (.07) | 0.14 (.09), 0.66 (.11) |
| Nelson (1999) | USA | -0.20 (.08) | 0.77 (.09) |
| Nelson (2003) | USA | -0.16 (.03), -0.12 (.04) | -0.06 (.08), -0.03 (.04) |
| Nelson & Moran (1995) | USA | -0.37 (.18), -0.04 (.09) | 0.69 (.12) |
| Niskanen (1962) | USA | -0.50 (.21) | -0.33 (.12) |
| Norman (1975) | USA | -0.87 (.11) | 0.36 (.03) |
| Norstrom (2005) | Sweden | -0.79 (.18), -0.90 (.27) |  |
| Ogwang & Cho (2009) | Canada | +0.05 (.62) | 0.19 (.10) |
| Ornstein & Hanssens (1985) | USA | -0.13 (.07) | -0.06 (.09) |
| Osoro (2005) | Tanzania | -0.31 (.05) | 1.04 (.30) |
| Ozguven (2004) | Turkey | -0.37 (.17) | 1.20 (.39) |
| Pagoulatos (1986) | USA | -0.28 (.18) |  |
| Pan (2006) | China | -0.90 (.90), -0.58 (.58) | 0.85 (.21) |
| Partanan (1991) | Kenya | -0.33 (.13) | 0.65 (.24) |
| Pearce (1985) | New Zealand | -0.15 (.07) | 0.85 (1.08) |
| Penm (1988) | Australia | -0.45 (.21) | 0.69 (.23) |
| Pierani & Tiezzi (2007) | Italy | -0.86 (.85), -0.39 (.38) | 1.90 (.92), 0.87 (.40) |
| Quek (1988) | Canada | -0.28 (.03), -0.16 (.22) | 0.77 (.15), 0.44 (.21) |
| Ruhm (2011) | USA | -0.28 (.50) | 0.09 (.04) |
| Sabuhoro et al. (1996) | Canada, ON | -0.72 (.05) | 1.61 (.10) |
| Salisu et al. (1997) | UK | -0.21 (.10), -0.32 (.16) | 0.37 (.07), 0.76 (.15) |
| Schweitzer et al. (1983) | USA | -0.27 (.29) | 0.43 (.38) |
| Selvanathan (1988) | UK | -0.13 (.06), -0.20 (.12) | 0.55 (.08), 0.41 (.18) |
| Selvanathan (1989) | UK | -0.25 (.07) | 0.27 (.09) |
| Selvanathan (1991) | Australia | -0.15 (.04) | 0.84 (.07) |
| Selvanathan (1991) | Canada | -0.26 (.17) | 0.71 (.16) |
| Selvanathan (1991) | Finland | -0.54 (.48) | 0.40 (.18) |
| Selvanathan (1991) | Japan | -0.25 (.45) | 1.43 (.31) |
| Selvanathan (1991) | New Zealand | -0.12 (.10) | 0.90 (.18) |
| Selvanathan (1991) | Norway | -0.14 (.25) | 0.34 (.12) |
| Selvanathan (1991) | Sweden | -0.35 (.10) | 0.22 (.18) |
| Selvanathan (1991) | UK | -0.13 (.06) | 0.52 (.08) |
| Selvanathan (1991) | USA | -0.11 (.09) | 0.71 (.12) |
| Selvanathan (1995) | Australia | -0.17 (.07) | 0.45 (.06) |
| Selvanathan (2004) | Australia | -0.16 (.03), -0.33 (.06) | 0.66 (.07), 0.46 (.05) |
| Selvanathan et al. (2005a) | Australia | -0.20 (.02) | 0.79 (.15) |
| Selvanathan et al. (2005a) | Canada | -0.22 (.02) | 0.67 (.13) |
| Selvanathan et al. (2005a) | Finland | -0.24 (.09) | 0.44 (.30) |
| Selvanathan et al. (2005a) | France | -0.06 (.03) | 0.66 (.97) |
| Selvanathan et al. (2005a) | New Zealand | -0.18 (.04) | 0.84 (.40) |
| Selvanathan et al. (2005a) | Sweden | -0.45 (.13) | 0.79 (.21) |
| Selvanathan et al. (2005a) | UK | -0.27 (.03) | 0.88 (.15) |
| Selvanathan et al. (2005b) | Australia | -0.65 (.19) | 0.77 (.07) |
| Selvanathan et al. (2005b) | Canada | -0.43 (.11) | 0.64 (.07) |
| Selvanathan et al. (2005b) | Finland | -0.81 (.58) | 0.42 (.17) |
| Selvanathan et al. (2005b) | France | -0.08 (.16) | 0.65 (.35) |
| Selvanathan et al. (2005b) | Japan | -0.33 (.28) | 1.31 (.10) |
| Selvanathan et al. (2005b) | New Zealand | -0.23 (.35) | 0.81 (.21) |
| Selvanathan et al. (2005b) | Norway | +0.09 (.27) | 0.40 (.11) |
| Selvanathan et al. (2005b) | Sweden | -0.29 (.26) | 0.81 (.25) |
| Selvanathan et al. (2005b) | UK | -0.67 (.14) | 0.80 (.09) |
| Selvanathan et al. (2005b) | USA | -0.29 (.09) | 0.81 (.07) |
| Selvanathan et al. (2007) | Australia | -0.20 (.06) | 0.78 (.08) |
| Selvanathan et al. (2007) | Canada | -0.25 (.06) | 0.65 (.07) |
| Selvanathan et al. (2007) | Finland | -0.64 (.39) | 0.43 (.18) |
| Selvanathan et al. (2007) | France | +0.01 (.16) | 0.80 (.31) |
| Selvanathan et al. (2007) | Japan | -0.03 (.19) | 1.30 (.12) |
| Selvanathan et al. (2007) | New Zealand | -0.13 (.11) | 0.88 (.20) |
| Selvanathan et al. (2007) | Norway | +0.08 (.12) | 0.37 (.10) |
| Selvanathan et al. (2007) | Sweden | -0.26 (.17) | 0.87 (.23) |
| Selvanathan et al. (2007) | UK | -0.31 (.06) | 0.84 (.09) |
| Selvanathan et al. (2007) | USA | -0.15 (.04) | 0.81 (.07) |
| Stone & Rowe (1958) | UK | -0.40 (.20), -0.53 (.22) | 0.52 (.26), 0.68 (.33) |
| Tegene (1990) | USA | -0.72 (.17) | 0.44 (.05) |
| Thom (1984) | Ireland | -0.68 (.24) | 0.80 (.19) |
| Tian & Liu (2011) | China | -0.29 (.06) |  |
| Treisman (2010) | Russia | -0.18 (.10) | -0.17 (.11) |
| Trolldal & Ponicki (2005) | USA | -0.07 (.03) | 0.19 (.05) |
| Uri (1986) | USA | -1.07 (.49) | 1.46 (.16) |
| Volland (2009) | Germany | -0.08 (.53) | 0.91 (.16) |
| Walsh (1982) | UK | -0.13 (.09) | 0.13 (.26) |
| Walsh & Walsh (1970) | Ireland | -0.17 (.20) | 0.78 (.10) |
| Wang et al. (1996) | USA | -0.37 (.03) |  |
| Wang et al. (1997) | China | -0.63 (.10) | 1.26 (.07) |
| Wette et al. (1993) | New Zealand | -1.10 (.48) | -0.01 (.05) |
| Wohlgenant (2011) | USA | +0.02 (.12), -0.14 (.13) | 0.85 (.21) |
| Yu & Chen (1998) | Canada, NB | -1.13 (.15) | 0.29 (.08) |
| Zereyesus (2010) | USA | -0.04 (.02), -0.91 (.46) | 1.01 (1.01) |
| Zhang & Casswell (1999) | New Zealand | -1.02 (.38) |  |
| Zhuk (2012) | USA | -0.18 (.08) | -0.02 (.04) |
| Zoltan (2006) | Hungary | -0.29 (.19) | 0.17 (.08) |

**Supplemental Table S2**. Wine Price and Income Elasticities

| **Study (year published)** | **Country** | **Wine price (se)** | **Wine income (se)** |
| --- | --- | --- | --- |
| Adrian & Ferguson (1987) | Canada | -0.61 (.18), -1.27 (0.08) | 0.70 (.17), 0.62 (.18) |
| Alley et al. (1992) | Canada, BC | -0.76 (.59) | 0.27 (.05) |
| Andrikopoulos et al. (1997) | Canada, ON | -0.51 (.39), -0.70 (.25) | 2.22 (.50), 6.04 (.50) |
| Andrikopoulos et al. (1997) | Canada, ON | -0.39 (.38), -0.35 (.22) |  |
| Andrikoupolos & Loizides (2000) | Cyprus | -0.24 (.40), -0.56 (.18) | 1.03 (.14), 0.70 (.31) |
| Angulo et al. (2001) | Spain | -1.04 (.52), -1.52 (.76) | 0.81 (.40), 1.14 (.57) |
| Ashton & Casswell (1987) | New Zealand | -1.37 (.66) | 0.54 (.40) |
| Asplund et al. (2007) | Sweden | -0.24 (.02) | 0.65 (.05) |
| Baltagi & Griffin (2006) | Sweden | -0.39 (.04), -2.73 (.73) | 0.20 (.04), 1.41 (.25) |
| Barnes (1984) | Canada | -1.05 (.09) | 0.05 (.02) |
| Bentzen et al. (1997) | Denmark | -0.29 (.16) | 0.89 (.39) |
| Bentzen et al. (1997) | Norway | -0.92 (.18) | 1.05 (.30) |
| Bentzen et al. (1997) | Sweden | -0.40 (.12) |  |
| Berggren (1997) | Sweden | -0.92 (.01) | 0.93 (.01) |
| Berggren (1997b) | Sweden | -0.57 (.14), -1.24 (.60) | -0.23 (.76), -0.04 (.15) |
| Blake & Nied (1997) | UK | -0.93 (.46), -0.82 (.41) | 1.50 (.75), 1.85 (.92) |
| Chang et al. (2002) | Australia | -0.82 (.20) | 1.25 (.35) |
| Clements & Johnson (1983) | Australia | -0.39 (.17), -0.43 (.17) | 0.78 (.28), 0.75 (.30) |
| Clements & Selvanathan (1988) | UK | -0.13 (.19) | 1.93 (.19) |
| Clements & Selvanathan (1988) | USA | -0.22 (.13) | 0.66 (.27) |
| Clements & Selvanathan (1991) | Australia | -0.37 (.17), -0.32 (.15) | 0.61 (.28), 0.61 (.28) |
| Clements et al. (1997) | Australia | -0.50 (.12) | 1.00 (.17) |
| Clements et al. (1997) | Canada | -0.44 (.13) | 1.05 (1.6) |
| Clements et al. (1997) | Finland | -1.78 (.29) | 1.32 (.24) |
| Clements et al. (1997) | New Zealand | -0.39 (.21) | 0.88 (.41) |
| Clements et al. (1997) | Norway | -0.12 (.02) | 1.48 (2.5) |
| Clements et al. (1997) | Sweden | -0.99 (.29) | 0.69 (.22) |
| Clements et al. (1997) | UK | -0.57 (.10) | 1.06 (.17) |
| Clements (2005) | Australia | -0.36 (.19) |  |
| Collis et al. (2010) | UK | -0.46 (.05), -0.54 (.04) | 0.90 (.35), 0.42 (.16) |
| Comanor & Wilson (1974) | USA | -0.84 (.45), -0.68 (.36) | 0.50 (1.39), 0.47 (1.3) |
| Coulson et al. (2001) | USA | -0.59 (.32) | 0.76 (.27) |
| Crawford & Tanner (1995) | UK | -1.40 (.31) |  |
| Crawford et al. (1999) | UK | -1.69 (.46) |  |
| Crooks (1989) | UK | -0.91 (.35) | 2.56 (.09) |
| Dahlstrom & Asberg (2009) | Sweden | -1.83 (.01), -2.25 (.02) | 0.80 (.01), 0.80 (.01) |
| Duffy (1982a) | UK | -0.97 (.46) | 2.41 (.29) |
| Duffy (1982b) | UK | -1.25 (.29), -1.14 (.27) | 1.96 (.38), 1.50 (.35) |
| Duffy (1983) | UK | -1.00 (.28), +0.87 (.22) | 2.22 (.21), 2.54 (.18) |
| Duffy (1987) | UK | -0.77 (.21), -1.13 (.26) | 1.70 (.17), 2.18 (.32) |
| Duffy (1990) | UK | -1.18 (.27), -1.26 (.24) | 1.98 (.39), 2.16 (.31) |
| Duffy (1991) | UK | -0.75 (.22) | 1.87 (.28) |
| Duffy (1995) | UK | -0.86 (.23), -0.49 (.25) | 2.53 (.52), 2.75 (.46) |
| Duffy (2001) | UK | -0.67 (.14), -0.83 (.19) | 1.12 (.09), 1.25 (.12) |
| Duffy (2003) | UK | -0.79 (.33) | 0.71 (.56) |
| Dyack & Goddard (2001) | Canada, ON | +0.43 (.29), -1.48 (.19) |  |
| Dyack & Goddard (2001) | Canada, ON | +0.33 (.11), -1.30 (.17) |  |
| Dyack & Goddard (2001) | Canada, ON | +0.03 (.02) | 1.14 (.13) |
| Eakins & Gallagher (2003) | Ireland | -0.80 (.40), -1.59 (.80) | 1.86 (.90), 2.33 (1.13) |
| Fang & Pan (2003) | China | -0.83, (.14), -1.36 (.23) | 1.39 (.26) |
| Gallet (2007) | USA | -0.41 (.05) | 1.60 (.17) |
| Gao et al. (1995) | USA | -0.40 (.13) | 5.03 (.72) |
| Godfrey (1988) | UK | -0.88 (.24) | 2.34 (.30) |
| Goldschmidt (1990) | Australia | -0.27 (.17) | 0.81 (1.16) |
| Gruenewald et al. (2006) | Sweden | -0.59 (.11) | -0.17 (.22) |
| Hagan & Waterson (1983) | UK | -0.40 (.14) | 1.25 (.34) |
| Heien & Pompelli (1989) | USA | -0.55 (.20) | 2.10 (.17) |
| Heien & Sims (2000) | Canada | -0.79 (.14), -0.66 (.12) | 2.06 (.62), 1.73 (.52) |
| Holm (1995) | Finland | -0.51 (.27) | 0.84 (.33) |
| Holm & Suoniemi (1992) | Finland | -2.31 (.54) |  |
| Janda et al. (2010) | Czech Republic | -1.09 (.16) | 0.76 (.03) |
| Jithitkulchai (2010) | USA | +0.62 (.48) | 0.04 (.04) |
| Johnson & Oksanen (1974) | Canada | -0.50 (.10), -1.30 (.26) | -0.01 (.08), -0.02 (.20) |
| Johnson & Oksanen (1977) | Canada | -0.68 (.10), -1.44 (.24) | 0.02 (.07), 0.04 (.14) |
| Johnson et al. (1992) | Canada | -0.88 (.18), -1.17 (.10) | 0.97 (.25), 2.19 (.06) |
| Johnson LW (1985) | UK | -0.18 (.11) | 1.43 (.16) |
| Jones (1989) | UK | -0.77 (.17), -0.94 (.25) | 1.46 (.37), 1.67 (.27) |
| Labys (1976) | USA | -0.44 (.37), -1.65 (.38) | 2.34 (.18), 3.34 (.28) |
| Lariviere et al. (2000) | Canada, ON | -0.83 (.49), -0.75 (.44) |  |
| Lau (1975) | Canada | -1.29 (.38) | 0.74 (.21) |
| Leong & Wang (1994) | USA, FL | -0.88 (1.44). -0.99 (1.62) | 1.00 (4.55) |
| Levi & Folwell (1995) | USA | -0.47 (.12), -0.16 (.06) | 2.25 (.30), 0.58 (.29) |
| Mangeloja & Pehkonen (2009) | Finland | -0.88 (.44) | 0.10 (.10) |
| McGuinness (1983) | UK | -0.17 (.15) | 1.11 (.48) |
| Meyerhoefer et al. (2005) | Romania | -1.17 (.05) | 0.85 (.04) |
| Moosa & Baxter (2002) | UK | -2.30 (.57) | 2.30 (.69) |
| Nelson (1990a) | USA | -1.86 (.68) | 1.05 (.65) |
| Nelson (1990b) | USA | -1.60 (.63) | 1.16 (.67) |
| Nelson (1997) | USA | -0.58 (.25), -0.52 (.23) | 0.20 (.14), 0.93 (.35) |
| Nelson (1999) | USA | -0.69 (.28) | 1.90 (.29) |
| Nelson (2003) | USA | -0.20 (.04), -0.28 (.06) | 1.93 (.08), 2.40 (.07) |
| Nelson & Moran (1995) | USA | -0.18 (.11), -0.08 (.14) | 0.98 (.30) |
| Niskanen (1962) | USA | -1.10 (.55) | 1.45 (.33) |
| Norstrom (2005) | Sweden | -0.57 (.18), -0.63 (.24) |  |
| Ogwang & Cho (2009) | Canada | -0.28 (.02) | 1.53 (.01) |
| Owen (1979) | Australia | -0.80 (.23) | 0.18 (.42) |
| Pan et al. (2006) | China | -1.36 (4.50), -0.93 (3.10) | 1.39 (.63) |
| Pearce (1985) | New Zealand | -0.35 (.16) | 1.14 (4.22) |
| Pierani & Tiezzi (2007) | Italy | -0.51 (.20), -0.37 (.17) | 1.90 (.22), 1.38 (.44) |
| Pierani & Tiezzi (2011) | Italy | -0.48 (.08), -1.10 (.18) |  |
| Quek (1988) | Canada | -0.58 (.12), -0.66 (.47) | 1.12 (.26), 1.26 (.33) |
| Sabuhoro et al. (1996) | Canada, ON | -0.99 (.04) | 0.26 (.09) |
| Salisu et al. (1997) | UK | -0.55 (.25), -1.09 (.49) | 0.77 (.19), 1.55 (.39) |
| Sam & Thompson (2008) | USA | -0.41 (.10) | 1.80 (.68) |
| Seale et al. (2003) | USA | -0.19 (.09) | 1.27 (.22) |
| Selvanathan (1988) | UK | -0.37 (.13), -0.49 (.14) | 1.23 (.20), 1.74 (.37) |
| Selvanathan (1989) | UK | -0.22 (.16) | 2.10 (4.12) |
| Selvanathan (1991) | Australia | -0.60 (.14) | 0.73 (.26) |
| Selvanathan (1991) | Canada | -0.16 (.35) | 0.97 (.25) |
| Selvanathan (1991) | Finland | -0.86 (.59) | 1.58 (.33) |
| Selvanathan (1991) | Japan | +0.80 (.33) | 0.29 (.36) |
| Selvanathan (1991) | New Zealand | -0.42 (.23) | 1.13 (.60) |
| Selvanathan (1991) | Norway | -0.07 (.23) | 1.44 (.19) |
| Selvanathan (1991) | Sweden | -0.87 (.15) | 0.48 (.19) |
| Selvanathan (1991) | UK | -0.40 (.13) | 1.31 (.21) |
| Selvanathan (1991) | USA | -0.05 (.14) | 0.63 (.27) |
| Selvanathan (1995) | Australia | -0.28 (.13) | 1.90 (.33) |
| Selvanathan et al. (2004) | Australia | -0.31 (.10), -0.39 (.12) | 0.83 (.25), 0.60 (.18) |
| Selvanathan et al. (2005a) | Australia | -0.43 (.10) | 1.00 (.19) |
| Selvanathan et al. (2005a) | Canada | -0.48 (.06) | 1.18 (.23) |
| Selvanathan et al. (2005a) | Finland | -0.78 (.16) | 1.52 (1.02) |
| Selvanathan et al. (2005a) | France | -0.05 (.01) | 0.88 (1.28) |
| Selvanathan et al. (2005a) | Japan | -0.13 (.03) | 0.63 (.46) |
| Selvanathan et al. (2005a) | New Zealand | -0.34 (.19) | 0.87 (.41) |
| Selvanathan et al. (2005a) | Norway | -0.14 (.01) | 1.23 (.11) |
| Selvanathan et al. (2005a) | Sweden | -0.32 (.11) | 0.46 (.12) |
| Selvanathan et al. (2005a) | UK | -0.35 (.08) | 0.67 (.11) |
| Selvanathan et al. (2005a) | USA | -0.27 (.05) | 1.06 (.23) |
| Selvanathan et al. (2005b) | Australia | -0.61 (.19) | 1.06 (.23) |
| Selvanathan et al. (2005b) | Canada | -0.57 (.12) | 1.35 (.12) |
| Selvanathan et al. (2005b) | Finland | -0.39 (.69) | 1.63 (.30) |
| Selvanathan et al. (2005b) | France | -0.09 (.14) | 0.85 (.24) |
| Selvanathan et al. (2005b) | Japan | -0.06 (.34) | 0.50 (.12) |
| Selvanathan et al. (2005b) | New Zealand | -0.78 (.22) | 1.15 (.51) |
| Selvanathan et al. (2005b) | Norway | -0.18 (.21) | 1.38 (.15) |
| Selvanathan et al. (2005b) | Sweden | -0.76 (.14) | 0.40 (.14) |
| Selvanathan et al. (2005b) | UK | -0.41 (.13) | 0.91 (.18) |
| Selvanathan et al. (2005b) | USA | -0.37 (.08) | 1.18 (.21) |
| Selvanathan et al. (2007) | Australia | -0.56 (.16) | 0.90 (.24) |
| Selvanathan et al. (2007) | Canada | -0.37 (.15) | 1.17 (.14) |
| Selvanathan et al. (2007) | Finland | -0.52 (.62) | 1.58 (.30) |
| Selvanathan et al. (2007) | France | -0.01 (.06) | 0.61 (.19) |
| Selvanathan et al. (2007) | Japan | +0.002 (.23) | 0.59 (.14) |
| Selvanathan et al. (2007) | New Zealand | -0.43 (.23) | 1.05 (.61) |
| Selvanathan et al. (2007) | Norway | -0.26 (.17) | 1.21 (.16) |
| Selvanathan et al. (2007) | Sweden | -0.49 (.13) | 0.37 (.18) |
| Selvanathan et al. (2007) | UK | -0.30 (.11) | 0.72 (.20) |
| Selvanathan et al. (2007) | USA | -0.25 (.08) | 1.12 (.22) |
| Tegene (1990) | USA | -1.20 (.40) | 0.67 (.18) |
| Thom (1984) | Ireland | -1.60 (3.3) | 1.39 (.39) |
| Trolldal & Ponicki (2005) | USA | -0.02 (.05) | 0.29 (.15) |
| Troncoso-Valverde (2004) | Chile | -0.48 (.18) | -1.72 (.64) |
| Uri (1986) | USA | -0.88 (.14) | 2.02 (.86) |
| Walsh (1982) | UK | -0.28 (.12) | 0.51 (.51) |
| Wang et al. (1996) | USA | +0.08 (.02), -0.83 (.02) | 1.00 (.01) |
| Wang et al. (1997) | China | -0.84 (.13) | 1.30 (.11) |
| Wette et al. (1993) | New Zealand | -1.10 (.35) | 0.30 (.43) |
| Wohlgenant (2011) | USA | -0.15 (.24), -0.66 (.11) | 7.57 (.78) |
| Yu & Chen (1998) | Canada, NB | -0.05 (.26) | 0.17 (.12) |
| Zereyesus (2010) | USA | -0.70 (.70), -0.76 (.76) | 0.79 (1.02) |
| Zhang & Casswell (1999) | New Zealand | -0.71 (.28) |  |

**Supplemental Table S3**. Spirits Price and Income Elasticities

| **Study (year published)** | **Country** | **Spirits price (se)** | **Spirits income (se)** |
| --- | --- | --- | --- |
| Adrian & Ferguson (1987) | Canada | -0.05 (.11), -0.96 (.17) | 0.89 (.11), 0.69 (.20) |
| Alley et al. (1992) | Canada, BC | -1.76 (8.0) | 0.26 (.04) |
| Andrikopoulos et al. (1997) | Canada, ON | -0.54 (.19), -0.34 (.22) | 0.08 (.13), 0.83 (.26) |
| Andrikopoulos et al. (1997) | Canada, ON | -0.51 (.07), -0.27 (.15) |  |
| Andrikoupolos & Loizides (2000) | Cyprus | -0.17 (3.1), -0.72 (.08) | 0.65 (.10), 1.454 (.15) |
| Angulo et al. (2001) | Spain | -1.04 (.52), -4.65 (2.32) | 0.91 (.46), 2.63 (1.32) |
| Ashton & Casswell (1987) | New Zealand | -1.25 (.33) | 0.16 (.26) |
| Asplund et al. (2007) | Sweden | -1.29 (.03) | 1.38 (.04) |
| Baltagi & Goel (1990) | USA | -0.63 (.19) |  |
| Baltagi & Griffin (1995) | USA | -0.20 (.03), -0.31 (.09) | 0.03 (.03), 0.32 (.06) |
| Baltagi & Griffin (1995) | USA | -0.69 (.21), -0.73 (.20) | 0.10 (.10), 0.78 (.14) |
| Baltagi & Griffin (2002) | USA | -0.51 (.15), -1.38 (.39) |  |
| Baltagi & Griffin (2002) | USA | -0.31 (.12), -1.62 (.60) |  |
| Baltagi & Griffin (2006) | Sweden | -0.26 (.09), -1.26 (.53) | 0.04 (.03), 0.18 (.24) |
| Baltagi & Li (2006) | USA | -0.68 (.04), -0.31 (.04) | 0.94 (.06), 0.61 (.08) |
| Barnes (1984) | Canada | -0.91 (.12) | 0.17 (.01) |
| Barsby & Marshall (1977) | USA | -1.06 (.45) | 1.23 (.32) |
| Bentzen et al. (1997) | Denmark | -0.89 (.18) | 0.46 (.36) |
| Bentzen et al. (1997) | Norway | -0.53 (.24) | 2.16 (.31) |
| Bentzen et al. (1997) | Sweden | -0.72 (.16) | 0.87 (.27) |
| Berggren (1997a) | Sweden | -1.03 (.01) | 1.00 (.01) |
| Berggren (1997b) | Sweden | -0.92 (.13), -1.97 (1.97) | 0.85 (.25), 0.68 (1.16) |
| Blake & Nied (1997) | UK | -1.32 (.66), -1.31 (.66) | 0.91 (.46), 1.16 (.58) |
| Clements & Johnson (1983) | Australia | -0.41 (.11), -0.74 (.14) | 1.97 (.27), 1.91 (.39) |
| Clements & Selvanathan (1988) | UK | -0.30 (.13) | 1.80 (.14) |
| Clements & Selvanathan (1988) | USA | -0.10 (.10) | 1.31 (.12) |
| Clements & Selvanathan (1991) | Australia | -0.83 (.11), -0.61 (.08) | 2.52 (.25), 2.51 (.25) |
| Clements et al. (1997) | Australia | -0.91 (.14) | 1.83 (.31) |
| Clements et al. (1997) | Canada | -0.52 (.06) | 1.25 (1.95) |
| Clements et al. (1997) | Finland | -1.78 (.09) | 1.32 (.24) |
| Clements et al. (1997) | New Zealand | -0.64 (.19) | 1.45 (.68) |
| Clements et al. (1997) | Norway | -0.12 (.08) | 1.55 (2.63) |
| Clements et al. (1997) | Sweden | -2.18 (.20) | 1.52 (.49) |
| Clements et al. (1997) | UK | -0.72 (.07) | 1.34 (.21) |
| Clements (2005) | Australia | -0.60 (.32) |  |
| Collis et al. (2010) | UK | -1.15 (.06).-0.90 (.09) | 0.55 (.21), 0.32 (.12) |
| Comanor & Wilson (1974) | USA | -0.30 (.61), -0.25 (.51) | 0.21 (.95), 0.18 (.81) |
| Coulson et al. (2001) | USA | -0.33 (.11) | 0.41 (.18) |
| Crawford & Tanner (1995) | UK | -1.81 (.27) |  |
| Crawford et al. (1999) | UK | -0.86 (.76) |  |
| Crooks (1989) | UK | -2.42 (.47) | 2.09 (.01) |
| Duffy (1982a) | UK | -1.06 (.39) | 1.64 (.21) |
| Duffy (1982b) | UK | -0.85 (.12), -0.84 (.15) | 1.57 (.18), 1.65 (.29) |
| Duffy (1983) | UK | -0.77 (.14), -0.79 (.14) | 1.67 (.33), 1.61 (.19) |
| Duffy (1987) | UK | -0.50 (.20), -0.85 (.24) | 1.42 (.15), 1.78 (.31) |
| Duffy (1990) | UK | -0.84 (.21), -0.79 (.19) | 1.42 (.32), 2.00 (.26) |
| Duffy (1991) | UK | -0.86 (.22) | 2.07 (.27) |
| Duffy (1995) | UK | -0.97 (.29), -0.94 (.22) | 2.53 (.51), 2.41 (.41) |
| Duffy (2001) | UK | -0.72 (.13), -0.72 (.17) | 1.13 (.08), 1.34 (.10) |
| Duffy (2002) | UK | -0.93 (4.25), -0.67 (1.04) | 1.17 (.44), 1.38 (.80) |
| Duffy (2003) | UK | -1.36 (.17) | 1.60 (.28) |
| Eakins & Gallagher (2003) | Ireland | -0.85 (.76), -0.75 (.67) | 0.86 (.22), 1.04 (.27) |
| Florkowski & McNamara (1992) | Poland | -0.62 (.34) | 1.48 (.59) |
| Gallet (1999) | USA | -1.35 (.30) | 0.67 (.27) |
| Gallet (2007) | USA | -0.23 (.05) | 0.43 (.10) |
| Gao et al. (1995) | USA | -0.25 (.15) | 1.21 (.36) |
| Godfrey (1988) | UK | -0.84 (.17) | 1.53 (.27) |
| Goel & Morey (1995) | USA | -0.13 (.04), -0.15 (.04) | 0.92 (.06), 0.88 (.06) |
| Goldschmidt (1990) | Australia | -0.54 (.11) | 2.39 (.43) |
| Gruenewald et al. (2006) | Sweden | -0.66 (.07) | -0.16 (.16) |
| Hagan & Waterson (1983) | UK | -0.95 (.26) | 1.56 (.30) |
| Heien & Pompelli (1989) | USA | -0.50 (.20) | 2.66 (.13) |
| Holm (1995) | Finland | -0.91 (3.00) | 0.78 (.42) |
| Holm & Suoniemi (1992) | Finland | -0.79 (.29) |  |
| Huang (2003) | UK | -1.31 (.15) | 0.69 (.12) |
| Janda et al. (2010) | Czech Republic | -1.21 (.09) | 0.47 (.01) |
| Jithitkulchai (2010) | USA | -0.71 (.94) | -0.01 (.02) |
| Johnson & Oksanen (1974) | Canada | -0.91 (.13), -1.60 (.23) | 0.23 (.06), 0.40 (.11) |
| Johnson & Oksanen (1977) | Canada | -1.13 (.13), -1.73 (.21) | 0.10 (.05), 0.16 (.08) |
| Johnson et al. (1992) | Canada | -0.63 (.24), +0.37 (.09) | 0.92 (.27), 1.02 (.05) |
| Johnson LW (1985) | UK | -0.30 (.15) | 1.90 (.20) |
| Jones (1989) | UK | -0.95 (.17), -0.79 (.16) | 1.14 (.31), 1.21 (.23) |
| Kochanowski & Heck (1987) | USA | -0.66 (.30) | 1.32 (.26) |
| Kong (2003) | Canada | -0.31 (.05) | 0.52 (.12) |
| Lariviere et al. (2000) | Canada, ON | -0.53 (.66), -0.48 (.60) |  |
| Lau (1975) | Canada | -1.17 (.33) | 0.59 (.17) |
| Leong & Wang (1994) | USA, FL | -0.52 (.09), -0.96 (.17) | 0.99 (.89) |
| Mangeloja & Pehkonen (2009) | Finland | -0.86 (.43) | 1.46 (.73) |
| McCornac & Filante (1984) | USA | -0.88 (.27) | 1.84 (.27) |
| McGuinness (1983) | UK | -0.38 (.21) | 1.54 (.59) |
| Meyerhoefer et al. (2005) | Romania | -1.13 (.05) | 0.78 (.03) |
| Nelson (1990a) | USA | -0.89 (.44) | 1.07 (.42) |
| Nelson (1990b) | USA | -0.83 (.37) | 1.06 (.40) |
| Nelson (1997) | USA | -0.82 (.19), -0.39 (.09) | 0.31 (.20), 1.50 (.16) |
| Nelson (1999) | USA | -0.11 (.05) | 1.06 (.12) |
| Nelson (2003) | USA | +0.06 (.04), -0.50 (.08) | 0.39 (.04), 0.51 (.06) |
| Nelson & Moran (1995) | USA | -0.62 (.30), -0.04 (.06) | 1.37 (.11) |
| Niskanen (1962) | USA | -1.33 (.29) | 0.61 (.12) |
| Norstrom (2005) | Sweden | -0.96 (.18), -0.81 (.24) |  |
| Ogwang & Cho (2009) | Canada | -1.23 (.01) | 0.61 (.01) |
| Ornstein & Hanssens (1985) | USA | -0.92 (.14) | 0.44 (.15) |
| Pearce (1985) | New Zealand | -0.32 (.17) | 1.31 (2.05) |
| Pierani & Tiezzi (2007) | Italy | -0.64 (1.75), -0.18 (.41) | 1.72 (1.78), 0.48 (.38) |
| Quek (1988) | Canada | -0.30 (.01), -0.66 (.26) | 1.20 (.14), 0.95 (.22) |
| Sabuhoro et al. (1996) | Canada, ON | -1.41 (.09) | 0.13 (.12) |
| Salisu et al. (1997) | UK | -1.52 (.31), -1.28 (.26) | 0.59 (.14), 0.88 (.21) |
| Sam & Thompson (2008) | USA | -0.41 (.10) | 1.80 (.68) |
| Schweitzer et al. (1983) | USA | -0.13 (.64) | 0.80 (.64) |
| Selvanathan (1988) | UK | -0.32 (.09), -0.79 (.11) | 1.82 (.14), 2.18 (.32) |
| Selvanathan (1989) | UK | -0.20 (.11) | 2.00 (.19) |
| Selvanathan (1991) | Australia | -0.61 (.12) | 1.94 (.28) |
| Selvanathan (1991) | Canada | -0.01 (.16) | 1.29 (.15) |
| Selvanathan (1991) | Finland | -0.73 (.21) | 1.29 (.09) |
| Selvanathan (1991) | Japan | -0.68 (.42) | 0.47 (.40) |
| Selvanathan (1991) | New Zealand | -0.52 (.30) | 1.18 (.51) |
| Selvanathan (1991) | Norway | -0.18 (.16) | 1.56 (.10) |
| Selvanathan (1991) | Sweden | -0.22 (.07) | 1.52 (.11) |
| Selvanathan (1991) | UK | -0.31 (.09) | 1.83 (.14) |
| Selvanathan (1991) | USA | -0.11 (.12) | 1.36 (.13) |
| Selvanathan (1995) | Australia | -0.10 (.09) | 1.72 (.38) |
| Selvanathan (2004) | Australia | -0.62 (.12), -1.30 (.25) | 2.47 (.27), 1.80 (.20) |
| Selvanathan et al. (2005a) | Australia | -0.64 (.08) | 1.80 (.35) |
| Selvanathan et al. (2005a) | Canada | -0.29 (.02) | 1.32 (.25) |
| Selvanathan et al. (2005a) | Finland | -0.30 (.03) | 1.29 (.87) |
| Selvanathan et al. (2005a) | France | -0.06 (.01) | 1.23 (1.78) |
| Selvanathan et al. (2005a) | Japan | -0.24 (.07) | 1.02 (.74) |
| Selvanathan et al. (2005a) | New Zealand | -0.40 (.12) | 1.45 (.69) |
| Selvanathan et al. (2005a) | Norway | -0.09 (.01) | 1.72 (.15) |
| Selvanathan et al. (2005a) | Sweden | -0.35 (.04) | 1.35 (.36) |
| Selvanathan et al. (2005a) | UK | -0.56 (.06) | 1.51 (.25) |
| Selvanathan et al. (2005a) | USA | -0.18 (.01) | 1.24 (.27) |
| Selvanathan et al. (2005b) | Australia | -0.68 (.16) | 1.93 (.25) |
| Selvanathan et al. (2005b) | Canada | -0.49 (.11) | 1.35 (.07) |
| Selvanathan et al. (2005b) | Finland | -1.43 (.72) | 1.24 (.12) |
| Selvanathan et al. (2005b) | France | -0.14 (.16) | 1.29 (.26) |
| Selvanathan et al. (2005b) | Japan | -0.44 (.24) | 1.26 (.29) |
| Selvanathan et al. (2005b) | New Zealand | -0.43 (.47) | 1.53 (.61) |
| Selvanathan et al. (2005b) | Norway | -0.21 (.19) | 1.64 (.11) |
| Selvanathan et al. (2005b) | Sweden | -0.84 (.22) | 1.15 (.12) |
| Selvanathan et al. (2005b) | UK | -0.80 (.14) | 1.44 (.16) |
| Selvanathan et al. (2005b) | USA | -0.26 (.14) | 1.35 (.12) |
| Selvanathan et al. (2007) | Australia | -0.57 (.14) | 1.92 (.28) |
| Selvanathan et al. (2007) | Canada | -0.26 (.09) | 1.35 (.09) |
| Selvanathan et al. (2007) | Finland | -0.67 (.39) | 1.27 (.12) |
| Selvanathan et al. (2007) | France | +0.06 (.08) | 1.48 (.21) |
| Selvanathan et al. (2007) | Japan | -0.29 (.21) | 1.02 (.30) |
| Selvanathan et al. (2007) | New Zealand | -0.53 (.33) | 1.27 (.56) |
| Selvanathan et al. (2007) | Norway | +0.02 (.11) | 1.73 (.10) |
| Selvanathan et al. (2007) | Sweden | -0.36 (.11) | 1.34 (.14) |
| Selvanathan et al. (2007) | UK | -0.56 (.10) | 1.56 (.17) |
| Selvanathan et al. (2007) | USA | -0.20 (.06) | 1.22 (.11) |
| Smith (1976) | USA | -1.63 (.72) | 1.70 (.46) |
| Swidler (1986) | USA | -1.41 (.41) |  |
| Tegene (1990) | USA | -1.09 (.20) | 1.81 (.17) |
| Thom (1984) | Ireland | -1.42 (.59) | 1.23 (.07) |
| Tian & Liu (2011) | China | -0.10 (.03) |  |
| Treisman (2010) | Russia | -0.29 (.13) | 0.15 (.11) |
| Trolldal & Ponicki (2005) | USA | -0.25 (.06) | 0.21 (.10) |
| Uri (1986) | USA | -1.21 (.58) | 0.23 (.60) |
| Walsh (1982) | UK | -0.47 (.16) | 1.20 (.60) |
| Walsh & Walsh (1970) | Ireland | -0.57 (.44) | 1.94 (.35) |
| Wang et al. (1996) | USA | -1.16 (.05), -0.85 (.04) | 0.96 (.01) |
| Wang et al. (1997) | China | -0.67 (.04) | 0.87 (.04) |
| Wette et al. (1993) | New Zealand | -0.50 (2.50) | 0.10 (.50) |
| Wohlgenant (2011) | USA | -0.19 (.07), -0.35 (.03) | 1.52 (.27) |
| Yu & Chen (1998) | Canada, NB | -1.13 (.44) | 0.29 (.17) |
| Zereyesus (2010) | USA | -1.21 (1.29), -1.28 (1.28) | 1.18 (1.18) |
| Zhang & Casswell (1999) | New Zealand | +0.05 (1.54) |  |

**Supplemental Table S4**. Alcohol Price and Income Elasticities

| **Study (year published)** | **Country** | **Alcohol price (se)** | **Alcohol income (se)** |
| --- | --- | --- | --- |
| Andrienko (2006) | Russia | -0.66 (.25) | 0.45 (.17) |
| Arranz (2009) | Spain | -0.61 (.18) | 0.53 (.20) |
| Ashton (1987) | New Zealand | -0.81 (.18) | 0.49 (.16) |
| Baltagi & Geihecker (2006) | Russia | -0.18 (.04), -0.15 (.03) |  |
| Bask & Melkersson (2004) | Sweden | -1.05 (.11) |  |
| Blake & Nied (1997) | UK | -1.52 (.76) | 1.08 (.54) |
| Calfee & Scheraga (1994) | France | -0.01 (.14) | 0.50 (.15) |
| Calfee & Scheraga (1994) | Germany | -0.44 (.17) | 0.11 (.22) |
| Calfee & Scheraga (1994) | Netherlands | -1.06 (.17) | 0.86 (.30) |
| Calfee & Scheraga (1994) | Sweden | -1.00 (.26) | 1.05 (.14) |
| Calfee & Scheraga (1994) | UK | -0.54 (.17) | 0.70 (.16) |
| Clements & Johnson (1983) | Australia | -0.58 (.10) | 0.97 (.21) |
| Clements et al. (1985) | UK | -0.61 (.20) | 0.87 (.39) |
| Clements & Selvanathan (1987) | Australia | -0.56 (.10) |  |
| Clements & Selvanathan (1987) | UK | -0.61 (.17) |  |
| Clements & Selvanathan (1987) | USA | -0.62 (.12) |  |
| Clements & Selvanathan (1991) | Australia | -0.59 (.07) | 1.00 (.09) |
| Clements et al. (1997) | Australia | -0.50 (.08) |  |
| Clements et al. (1997) | Canada | -0.42 (.27) |  |
| Clements et al. (1997) | Finland | -1.35 (.25) |  |
| Clements et al. (1997) | New Zealand | -0.44 (.20) |  |
| Clements et al. (1997) | Norway | -0.08 (.14) |  |
| Clements et al. (1997) | Sweden | -1.43 (.46) |  |
| Clements et al. (1997) | UK | -0.54 (.08) |  |
| Decker & Schwartz (2000) | USA | -0.76 (.04) | 0.19 (.01) |
| Duffy (1982b) | UK | -0.64 (.14) | 1.40 (.16) |
| Duffy (1987) | UK | -0.62 (.20) | 1.24 (.17) |
| Duffy (1990) | UK | -0.51 (.14), -0.52 (.12) | 1.14 (.19), 1.26 (1.26) |
| Duffy (1991) | UK | -0.45 (.08) | 1.07 (.11) |
| Duffy (2001) | UK | -0.54 (.08), -0.44 (.10) | 0.94 (.12), 1.03 (.15) |
| Duffy (2002) | UK | -0.97 (3.93), -1.06 (1.92) | 0.69 (.20), 0.71 (.28) |
| Fanelli (2008) | UK | -1.23 (.42) | 3.80 (.56) |
| Fang (2011) | Multiple | -0.49 (.16) | 0.51 (.18) |
| Grabowki (1976) | USA | -0.39 (.15) | 0.34 (.07) |
| Gruber et al. (2002) | Canada | -1.34 (.16) |  |
| Hagan (1983) | UK | -0.79 (.19) | 0.59 (.14) |
| Holm & Suoniemi (1992) | Finland | -0.80 (.12) | 1.02 (.29) |
| Hsieh (1999) | Taiwan | -0.58 (.11) | 0.34 (.10) |
| John (2005) | India | -0.87 (.08), -1.03 (.04) | 0.50 (.19), 0.72 (.28) |
| Koskal (2012) | USA | -1.34 (.20), -1.56 (.25) | 0.41 (.06), 0.41 (.06) |
| Lee (2007) | Taiwan | -0.58 (.13) | 0.81 (.33) |
| Lee et al. (2010a) | Taiwan | -0.77 (.05) | 1.09 (.06) |
| Lee et al. (2010b) | Taiwan | -0.37 (.12) | 1.52 (.56) |
| Leppanen et al. (2001) | Austria | +0.36 (.12) | 0.52 (.11) |
| Leppanen et al. (2001) | Belgium | -0.24 (.09) | 0.58 (.12) |
| Leppanen et al. (2001) | Denmark | -0.79 (.12) | 0.51 (.12) |
| Leppanen et al. (2001) | Finland | -0.57 (.12) | 0.78 (.05) |
| Leppanen et al. (2001) | France | -0.32 (.12) | 1.02 (.18) |
| Leppanen et al. (2001) | Greece | -0.62 (.34) | -0.26 (.65) |
| Leppanen et al. (2001) | Ireland | -0.78 (.17) | 0.36 (.14) |
| Leppanen et al. (2001) | Italy | +0.34 (.23) | -0.12 (.40) |
| Leppanen et al. (2001) | Netherlands | -0.53 (.22) | 0.90 (.24) |
| Leppanen et al. (2001) | Norway | -1.49 (.22) | 0.74 (.17) |
| Leppanen et al. (2001) | Portugal | -0.42 (.15) | 0.55 (.18) |
| Leppanen et al. (2001) | Spain | -0.35 (.18) | 0.95 (.22) |
| Leppanen et al. (2001) | Sweden | -1.63 (.20) | 1.44 (.21) |
| Leppanen et al. (2001) | UK | -0.48 (.11) | 0.48 (.18) |
| Leskinin (1976) | Finland | -0.77 (.18) |  |
| Levy & Sheflin (1983) | USA | -0.49 (.11) | 0.38 (.13) |
| Madden (1993) | Ireland | -0.25 (.13) | 0.39 (.20) |
| Manning et al. (1995) | USA | -1.19 (.25) | 0.28 (.03) |
| McCarthy (1977) | Ireland | -0.80 (.11) | -1.24 (.17) |
| McGuinness (1980) | UK | -0.26 (.12) | 0.46 (.26) |
| Menon (2012) | Italy | -1.24 (.27) | 0.93 (.22) |
| Nelson (1997) | USA | -0.52 (.13) | 0.21 (.14) |
| Nelson (1999) | USA | -0.36 (.09) | 0.16 (.14) |
| Nelson (2003) | USA | -0.07 (.03), -0.23 (.04) | 0.38 (.03), 0.32 (.03) |
| Nelson (2010) | Multiple | -0.30 (.02) | 0.23 (.03) |
| Nelson & Moran (1995) | USA | -0.53 (.10) | 0.41 (.11) |
| Nelson & Young (2001) | Multiple | -0.52 (.05) | 0.38 (.09) |
| Pearce (1985) | New Zealand | -0.40 (.19) |  |
| Pierani (2007) | Italy | -0.47 (.38), -0.26 (.19) | 2.17 (.60), 1.17 (.20) |
| Pierani (2009) | Italy | -0.61 (.81) | 2.11 (1.32) |
| Quek (1988) | Canada | -0.51 (.23) | 0.79 (.25) |
| Saffer (2002) | Multiple | -0.19 (.09) |  |
| Sevanathan (1988) | UK | -0.50 (.12) | 1.10 (.15) |
| Sevanathan (1989) | UK | -0.45 (.17) | 1.13 (.29) |
| Sevanathan (2004) | Australia | -0.61 (.19) | 0.72 (.21) |
| Sevanathan (2005b) | Australia | -0.72 (.15) | 1.11 (.31) |
| Sevanathan (2005b) | Canada | -0.83 (.18) | 0.95 (.17) |
| Sevanathan (2005b) | Denmark | -0.81 (.22) | 0.64 (.23) |
| Sevanathan (2005b) | Finland | -0.64 (.12) | 0.94 (.21) |
| Sevanathan (2005b) | France | -0.17 (.11) | 0.08 (.12) |
| Sevanathan (2005b) | Ireland | -0.22 (.16) | 0.61 (.18) |
| Sevanathan (2005b) | Italy | -0.13 (.09) | 0.76 (.24) |
| Sevanathan (2005b) | Spain | -0.49 (.12) | 0.94 (.57) |
| Sevanathan (2005b) | Sweden | -0.53 (.22) | 1.11 (.27) |
| Sevanathan (2005b) | UK | -0.11 (.38) | 0.80 (.26) |
| Sevanathan (2005b) | USA | -0.19 (.13) | 0.74 (.27) |
| Sevanathan (2006) | Australia | -0.53 (.13) |  |
| Sevanathan (2006) | Canada | -0.66 (.14) |  |
| Sevanathan (2006) | Finland | -0.70 (.12) |  |
| Sevanathan (2006) | Japan | -0.76 (.29) |  |
| Sevanathan (2006) | New Zealand | -0.41 (.16) |  |
| Sevanathan (2006) | Sweden | -0.51 (.12) |  |
| Sevanathan (2006) | UK | -0.76 (.16) |  |
| Sevanathan (2006) | USA | -0.46 (.17) |  |
| Shi (2011) | China | -0.14 (.03) | 0.06 (.02) |
| Taube (1990) | USA | -0.53 (.16) | 1.28 (1.29) |
| Taube (1991) | USA | -0.29 (.13) | 1.18 (1.12) |
| Tiffin (2011) | UK | -1.12 (.42), -0.42 (.16) |  |
| West (2009) | USA | -1.19 (.77) |  |
| Wette et al. (1993) | New Zealand | -2.00 (.57) | 0.01 (.01) |
| Wilkinson (1987a) | USA | -0.05 (.04) | 0.38 (.08) |
| Wilkinson (1987b) | USA | -0.44 (.47) | 0.36 (.09) |
| Yen (1994) | USA | -0.34 (.04) | 0.40 (.06) |
| Yu & Abler (2010) | China | -0.78 (.34) | 0.15 (.19) |
| Zhang & Casswell (1999) | New Zealand | -2.08 (.45) |  |
