# Supplementary material for: Meta-analysis of alcohol price and income elasticities – with corrections for publication bias
Source: Health Econ Rev. 2013 Jul 24;3:17. doi: 10.1186/2191-1991-3-17 (PMC3722038; doi:10.1186/2191-1991-3-17)
Supplement: Additional file 2: Table S1 — Beer price and income elasticities. Table S2. Wine price and income elasticities. Table S3. Spirits price and income elasticities. Table S4. Alcohol price and income elasticities. [file 2191-1991-3-17-S2.docx]

SUPPLEMENTAL APPENDIX

**ALCOHOL STUDIES BIBLIOGRAPHY**

**CONTENTS:**

**IA. STUDIES INCLUDED IN META-ANALYSIS (182 studies) – page 1**

**IIB. GALLET & FOGARTY STUDIES EXCLUDED [reason] (58 studies) – page 12**

**IC. OTHER EXCLUDED STUDIES FROM META-ANALYSIS [reason] (115 studies) – page 16**

**IIA. ADULT DRINKING: SURVEY STUDIES (19 studies) – page 23**

**IIB. EXCLUDED STUDIES: ADULTS [reason] (29 studies) – page 24**

**IIC. LIVER CIRRHOSIS MORTALITY STUDIES (9 studies) – pg. 26**

**IID. EXCLUDED STUDIES : CIRRHOSIS & MORTALITY [reason] (42 studies) – page 26**

**IIIA. GENDER-RELATED DRINKING SURVEY STUDIES: ADULTS (15 studies) – page 30**

**IIIB. EXCLUDED STUDIES: GENDER – ADULTS [reason] (39 studies) – page 31**

**IIIC. GENDER-RELATED DRINKING SURVEY STUDIES: YOUNG ADULTS (8 studies) – pg. 34**

**IIID. EXCLUDED STUDIES: GENDER – YOUNG ADULTS [reason] (62 studies) – page 34**

**TOTAL STUDIES: 578**

April 15, 2013

Working Draft

**IA. STUDIES INCLUDED IN META-ANALYSIS**

(*New Studies compared to Gallet and Fogarty)

1. Adrian, M. and Ferguson, B.S. (1987). Demand for domestic and imported alcohol in Canada. Applied Economics, 19, 531-40.

2. Alley, A.G., D.G. Ferguson, and Stewart, K.G. (1992). An almost ideal demand system for alcoholic beverages in British Columbia. Empirical Economics, 17, 401-18.

3. *Andrienko, Y. and Nemtsov, A. (2006). Estimation of individual demand for alcohol. Working paper, Centre for Economic and Financial Research, New Economic School.

4. Andrikopoulos, A.A., Brox, J.A., and Carvalho, E. (1997). The demand for domestic and imported alcoholic beverages in Ontario, Canada: A dynamic simultaneous equation approach. Applied Economics, 29, 945-53.

5. Andrikopoulos, A.A. and Loizides, J. (2000). The demand for home-produced and imported alcoholic beverages in Cyprus: The AIDS approach. Applied Economics, 32, 1111-19.

6. Angulo, A.M., Gil, J.M., and Gracia, A. (2001). The demand for alcoholic beverages in Spain. Agricultural Economics, 26, 71-83.

7. *Arranz, J.M. and Gil, A.I. (2009). Traffic accidents, deaths and alcohol consumption. Applied Economics, 41, 2583-95.

8. *Ashton, T. and S. Casswell (1987). Alcohol taxation as a public health policy: The New Zealand experience. Community Health Studies, 11, 108-19.

9. *Asplund, M., R. Friberg, and Wilander, F. (2007). Demand and distance: Evidence on cross-border shopping. Journal of Public Economics, 91, 141-57.

10. *Baltagi, B.H. and Geishecker, I. (2006). Rational alcohol addiction: Evidence from the Russian longitudinal monitoring survey. Health Economics, 15, 893-914.

11. Baltagi, B.H. and Goel, R.K. (1990). Quasi-experimental price elasticity of liquor demand in the United States: 1960-93. American Journal of Agricultural Economics, 72, 451-54.

12. Baltagi, B.H. and Griffin, J.M. (1995). A dynamic demand model for liquor: The case of pooling. Review of Economics and Statistics, 77, 545-54.

13. Baltagi, B.H. and Griffin, J.M. (2002). Rational addiction to alcohol: Panel data analysis of liquor consumption. Health Economics, 11, 485-91.

14. *Baltagi, B.H. and Li. D (2006). Prediction in the panel data model with spatial correlation: The case of liquor. Spatial Economic Analysis, 1, 175-85.

**182**. Baltagi, B.H. and Griffin, J.M. (2006). Swedish liquor consumption: New evidence on taste change, in B.H. Baltgi (ed.), Panel data econometrics: Theoretical contributions and empirical applications. Amsterdam: Elsevier, pp. 167-92. **OUT-OF-ORDER**.

15. *Barnes, K.G. (1984). Per capita consumption of alcohol: An examination of the relative importance of advertising and other factors. Working paper, Business Administration, Memorial University of Newfoundland.

16. Barsby, S.L. and Marshall, G.L. (1977). Short-term consumption effects of a lower minimum alcohol-purchasing age. Journal of Studies on Alcohol, 38, 1665-79.

17. Bask, M. and Melkersson, M. (2004). Rationally addicted to drinking and smoking? Applied Economics, 36, 373-81.

18. Bentzen, J., Smith, V., and N. Nannerup (1997). Alcohol consumption and drunken driving in the Scandinavian countries. Working paper no. 97-1, Department of Economics, Aarhus School of Business.

19. *Berggren, F. (1997a). The demand for alcohol in Sweden 1985-1995: A system approach. In: F. Berggren, Essays on the demand for alcohol in Sweden: Review and applied demand studies (pp. 115-43). Lund Economic Studies number 71, Lund University.

20. *Berggren, F. (1997b). Swedes and alcohol: An empirical analysis of rational addiction model. In: F. Berggren, Essays on the demand for alcohol in Sweden: Review and applied demand studies (pp. 165-79). Lund Economic Studies number 71, Lund University.

21. Blake, D. and Nied, A. (1997). The demand for alcohol in the United Kingdom, Applied Economics, 29, 1655-72.

22. Calfee, J.E. and Scheraga, C. (1994). The influence of advertising on alcohol consumption: A literature review and an econometric analysis of four European nations. International Journal of Advertising, 13, 287-310.

23. Chang, H-S., Griffith, G., and Bettington, N. (2002). The demand for wine in Australia using a systems approach: Industry implications. Agribusiness Review, 10, 1-13.

24. *Chetty, R., A. Looney, and K. Kroft (2009). Salience and taxation: Theory and evidence. American Economic Review, 99, 1145-77.

25. Clements, K.W. and Johnson, L.W. (1983). The demand for beer, wine, and spirits: A systemwide approach. Journal of Business, 56, 273-304.

26. *Clements, K.W., McLeod, P.B., and Selvanathan, E.A. (1985). Does advertising affect drinking and smoking? Working paper, Department of Economics, University of Western Australia.

27. Clements, K.W. and Selvanathan, E.A. (1987). Alcohol consumption. In: H. Theil and K. Clements (eds.), Applied demand analysis: Results from system-wide approaches (pp. 185-264). Cambridge: Ballinger.

28. Clements, K.W. and Selvanathan, E.A. (1988). The Rotterdam demand model and its application in marketing. Marketing Science, 7, 60-75.

29. Clements, K.W. and Selvanathan, S. (1991). The economic determinants of alcohol consumption. Australian Journal of Agricultural Economics, 35, 209-31.

30. Clements, K.W., Yang, W., and Zheng, S.H. (1997). Is utility additive? The case of alcohol. Applied Economics, 29, 1163-67.

31. *Clements, K.W. and Daryl, M. (2005). The economics of marijuana consumption. In: S. Selvanathan and E.A. Selvanathan, The demand for alcohol, tobacco and marijuana: International evidence (pp. 243-67). Aldershot, UK: Ashgate.

32. *Collis, J., Grayson, A., and Johal, S. (2010). Econometric analysis of alcohol consumption in the UK. HMRC working paper 10. London: HM Revenue & Customs.

33. Comanor, W.S. and Wilson, T.A. (1974). Advertising and market power. Cambridge: Harvard University Press.

34. Coulson, N.E., Moran, J.R., and Nelson, J.P. (2001). The long-run demand for alcoholic beverages and the advertising debate: A cointegration analysis. In: M.R. Baye and J.P. Nelson (eds.), Advances in Applied Microeconomics: Advertising and Differentiated Products, Vol. 10 (pp. 31-54). Amsterdam: JAI Press.

35. Crawford, I. and Tanner, S. (1995). Bringing it back home: Alcohol taxation and cross-border shopping. Fiscal Studies, 16, 94-114.

36. Crawford, I., Smith, Z., and Tanner, S. (1999). Alcohol taxes, tax revenues, and the single European market. Fiscal Studies, 20, 287-304.

37. Crooks. E. (1989). Alcohol consumption and taxation. London: The Institute for Fiscal Studies.

38. *Dahlstrom, T. and Asberg, A. (2009). Determinants of demand for wine – Price sensitivity and perceived quality in a monopoly setting. Working paper 182, Jonkoping International Business School, Royal Institute of Technology.

39. Decker, S.L. and Schwartz, A.E. (2000). Cigarettes and alcohol: Substitutes or complements? NBER working paper 7535. National Bureau of Economic Research.

40. Duffy, M. (1982a). A case study in econometric forecasting for alcoholic drinks. Omega: The International Journal of Management Science, 10, 597-611.

41. Duffy, M. (1982b). The effect of advertising on the total consumption of alcoholic drinks in the United Kingdom: Some econometric estimates. Journal of Advertising, 1, 105-17.

42. Duffy, M. (1983). The demand for alcoholic drink in the United Kingdom, 1963-78. Applied Economics, 15, 125-140.

43. Duffy, M. (1987). Advertising and the inter-product distribution of demand: A Rotterdam model approach. European Economic Review, 31, 1051-70.

44. Duffy, M. (1990). Advertising and alcoholic drink demand in the UK: Some further Rotterdam model estimates. International Journal of Advertising, 9, 247-57.

45. Duffy, M. (1991). Advertising and the consumption of tobacco and alcoholic drink: A system-wide analysis. Scottish Journal of Political Economy, 38, 369-85.

46. Duffy, M. (1995). Advertising in demand systems for alcoholic drinks and tobacco: A comparative study. Journal of Policy Modeling, 17, 557-77.

47. Duffy, M. (2001). Advertising in consumer allocation models: Choice of functional form. Applied Economics, 33, 437-56.

48. Duffy, M. (2002). On the estimation of an advertising-augmented, cointegrating demand system. Economic Modelling, 20, 181-206.

49. *Duffy, M. (2003). Advertising and food, drink and tobacco consumption in the United Kingdom: A dynamic demand system. Agricultural Economics 28, 51-70.

50. *Dyack, B. and Goddard, E. (2001). The rise of red and the wane of white: Wine demand in Ontario Canada. Working paper, Department of Rural Economy, University of Alberta.

51. Eakins, J.M. and Gallagher, L.A. (2003). Dynamic almost ideal demand systems: An empirical analysis of alcohol expenditure in Ireland. Applied Economics 35, 1025-36.

52. *Fanelli, L. and Mazzocchi, M. (2008). Rational addiction, cointegration and tobacco and alcohol demand. Working paper, Department of Statistics, University of Bologna.

53. *Fang, C. and Pan, S. (2003). Liquor and beverage consumption in China: A censored demand system approach.Working paper, Food and Agriculture Organization, United Nations.

54. Florkowski, W.J. and McNamara, K.T. (1992). Policy implications of alcohol and tobacco demand in Poland. Journal of Policy Modeling, 14, 93-8.

55. Fung, J.C. (2011). The international nature of alcohol as determined by cross-country analysis of demographics and pricing, 1995-2002. Undergraduate Economic Review, 7, Article 16, 1-42.

56. Gallet, C.A. (1999). Gradual switching regression estimates of alcohol demand elasticities. Applied Economics Letters, 6, 377-79.

57. Gallet, C.A. and Eastman, H.S. (2007). The impact of smoking bans on alcohol demand. Social Science Journal, 44, 664-76.

58. Gallet, C.A. and List, J.A. (1998). Elasticities of beer demand revisited. Economics Letters, 61, 67-71.

59. Gao, X.M., Wailes, E.J., and Cramer, G.L. (1995). A microeconometric model analysis of US consumer demand for alcoholic beverages. Applied Economics, 27, 59-69.

60. Godfrey, C. (1988). Licensing and the demand for alcohol. Applied Economics, 20, 1541-58.

61. Goel, R.K. and Morey, M.J. (1995). The interdependence of cigarette and liquor demand. Southern Economic Journal, 62, 451-59.

62. *Goldschmidt, P.S. (1990). Economic aspects of alcohol consumption in Australia: Parts 1 and 2. Working paper, Department of Economics, University of Western Australia.

63. Grabowski, H.G (1976). The effects of advertising on the interindustry distribution of demand. Explorations in Economic Research, 3, 21-75.

64. Gruber, J., Sen, A., and Stabile, M. (2002). Estimating price elasticities when there is smuggling: The sensitivity of smoking to price in Canada. Journal of Health Economics, 22, 821-42.

65. Gruenewald, P.J., Ponicki, W.R., Holder, H.D., and Romelsjo, A. (2006). Alcohol prices, beverage quality, and the demand for alcohol: Quality substitutions and price elasticities. Alcoholism: Clinical and Experimental Research, 30, 96-105.

66. Hagan, L.W. and Waterson, M.J. (1983). The impact of advertising on the United Kingdom alcoholic drink market. London: Advertising Association.

67. Heien, D. and Pompelli, G. (1989). The demand for alcoholic beverages: Economic and demographic effects. Southern Economic Journal, 55, 759-70.

68. Heien, D. and Sims, E.N. (2000). The impact of the Canada-United States Free Trade Agreement on U.S. wine exports. American Journal of Agricultural Economics 82, 173-82.

69. Hogarty, T.F. and Elzinga, K.G. (1972). The demand for beer. Review of Economics and Statistics, 54, 195-98.

70. Holm, P. (1995). Alcohol content and demand for alcoholic beverages: A system approach. Empirical Economics, 20, 75-92.

**177**. Holm, P. and Suoniemi, I. (1992). Empirical application of optimal commodity tax theory to taxation of alcoholic beverages. Scandinavian Journal of Economics, 94, 85-101. **OUT-OF-ORDER**.

71. Hsieh, C-R., Shi, M-S., and Lin, C-F.J. (2005). The impact of trade liberalization on alcohol consumption in Taiwan. In: B. Lindgren and M. Grossman (eds.), Substance use: Individual behaviour, social interactions, markets and politics, vol. 16 (pp. 413-32). Amsterdam: Elsevier.

72. Huang, C-D. (2003). Econometric models of alcohol demand in the United Kingdom. Working paper no. 140, Customs and Excise, HM Treasury.

73. *Janda, K., Mikolasek, J., and Netuka, M. (2010). Complete almost ideal demand system approach to the Czech alcohol demand. Agricultural Economics – Czech, 56, 421-34.

74. *Jithitikulchai, T. (2010). Alcohol consumption: Tax instrumental variables in quadratic almost ideal demand system (QUAIDS) with clustered data. International Conference on Applied Economics – ICOAE 2010, 363-71.

75. *John, R.M. (2005). Price elasticity estimates for tobacco and other addictive goods in India. Working paper No.WP-2005-003, Indira Gandi Institute of Development Research.

76. Johnson, J.A. and Oksanen, E.H. (1974). Socio-economic determinants of the consumption of alcoholic beverages. Applied Economics, 6, 293-301.

77. Johnson, J.A. and Oksanen, E.H (1977). Estimation of demand for alcoholic beverages in Canada from pooled time series and cross sections. Review of Economics and Statistics, 59, 113-18.

78. Johnson, J.A., Oksanen, E.H., Veall, M.R., and Fretz, D. (1992). Short-run and long-run elasticities for Canadian consumption of alcoholic beverages: An error-correction mechanism/cointegration approach. Review of Economics and Statistics, 74, 64-74.

79. Johnson, L.W. (1985). Alternative econometric estimates of the effect of advertising on the demand for alcoholic beverages in the United Kingdom. International Journal of Advertising, 4, 19-25.

80. Jones, A.M. (1989). A systems approach to the demand for alcohol and tobacco. Bulletin of Economic Research, 41, 85-105.

81. *Keane, J.G. (1965). An analysis of per capita beer demand in the United States, 1935-1963. Unpublished Ph.D. dissertation, Department of Economics, University of Pittsburgh.

82. *Kochanowski, P. and Heck, T. (1987). Shifts in the demand for alcohol: Some empirical findings. Unpublished paper, Department of Economics, Indiana University at South Bend.

83. *Koksal, A. (2012). Three essays on the interdependence between cigarette and alcohol consumption. Unpublished Ph.D. dissertation, Department of Economics, North Carolina State University.

84. *Kong, Q. (2003). Does advertising promote spirits consumption in Canada? Working paper, University of Western Australia.

85. Labys, W. (1976). An international comparison of price and income elasticities for wine consumption. Australian Journal of Agricultural Economics, 20, 33-6.

86. Lariviere, E., Larue, B., and Chalfant, J. (2000). Modeling the demand for alcoholic beverages and advertising specifications. Agricultural Economics, 22, 147-62.

87. Lau, H-H. (1975). Cost of alcoholic beverages as a determinant of alcohol consumption. In: R. Gibbons, et al. (eds.), Research advances in alcohol and drug problems, vol. 2 (pp. 211-45). New York: John Wiley.

88. Lee, B. and Tremblay, V.J. (1992). Advertising and the US market demand for beer. Applied Economics, 24, 69-76.

89. *Lee, J-M. (2007). They synergistic effect of cigarette taxes on the consumption of cigarettes, alcohol and betel nuts. BMC Public Health 7, 121. DOI: 10.1186/1471-2458-7-121.

90. *Lee, J-M., Chen, M-G., Hwang, T-C., and Yeh, C-Y. (2010). Effect of cigarette taxes on the consumption of cigarettes, alcohol, tea and coffee in Taiwan. Public Health, 124, 429-36.

91. Lee, J-M., Chen, S-H., Liu, H-H., et al. (2010). Effects of health risk information on addictive goods consumption: A case of tobacco, alcohol, and betel nuts in Taiwan. Contemporary Economic Policy, 28, 406-13.

92. *Leong, S. and Wang, X. ( 1994). Consumer demand for alcoholic beverages in Florida: Consequences of a check-off program. Proceedings of the Florida State Horticulture Society, 107, 312-14.

93. Leppanen, K., Sullstrom,R., and Suoniemi, I. (2001). Effects of economics factors on alcohol consumption in 14 European countries. Nordisk Alkohol & Narkotikatidskrift, 18, 100-16. The longer report also was used: Leppanen, K., Sullstrom, R., and Suoniemi, I. (2001). The consumption of alcohol in fourteen European Nations: A comparative econometric analysis. Helsinki: STAKES.

94. *Leskinen, E. and Terasvirta, T. (1976). Forecasting the consumption of alcoholic beverages in Finland: A Box-Jenkins approach. European Economic Review, 8, 349-69.

95. Levi, A.E. and Fowell, R.J. (1995). U.S. demand for imported wine. Journal of International Food & Agribusiness Marketing, 7, 79-91.

96. Levy, D. and Sheflin, N. (1983). New evidence on controlling alcohol use through price. Journal of Studies on Alcohol, 44, 929-37.

**181.** Madden, D. (1993). A new set of consumer demand estimates for Ireland, Economic and Social Review, 24, 101-23. . **OUT-OF-ORDER**

97. *Mangeloja, E. and Pehkonen (2009). Availability and consumption of alcoholic beverages: Evidence from Finland. Applied Economics Letters, 16, 425-29.

98. Manning, W.G, Blumberg, L., and Moulton, L.H. (1995). The demand for alcohol: The differential response to price. Journal of Health Economics, 14, 123-48.

99. *McCarthy, C. (1977). Estimates of a system of demand equations using alternative commodity classifications of Irish data, 1953-1974. Economic and Social Review, 8, 201-11.

100. McCornac, D.C. and Filante, R.W. (1984). The demand for distilled spirits: An empirical investigation. Journal of Studies on Alcohol, 45, 176-78.

101. McGuinness, T. (1980). An econometric analysis of total demand for alcoholic beverages in the U.K., 1956-75. Journal of Industrial Economics, 29, 85-109.

102. McGuinness, T (1983). The demand for beer, spirits and wine in the UK, 1956-79. In: M. Grant, M. Plant, and A. Williams (eds.), Economics and Alcohol: Consumption and Controls (pp. 238-242). New York: Gardner.

103. *Menon, M., Perali, P., and Piccoli, L. (2012). The passive drinking effect: A collective demand application. Working paper, University of Verona.

104. *Meyerhoefer, C.D., Ranney, C.K., and Sahn, D.E. (2005). Consistent estimation of censored systems using panel data. American Journal of Agricultural Economics, 87, 660-72.

105. Moosa, I.A. and Baxter, J.L. (2002). Modelling the trend and seasonals within an AIDS model of the demand for alcoholic beverages in the United Kingdom. Journal of Applied Econometrics, 17, 95-106.

106. Nelson, J.P. (1990a). State monopolies and alcoholic beverage consumption. Journal of Regulatory Economics, 2, 83-98.

107. *Nelson, J.P. (1990b). Effect of regulation on alcoholic beverage consumption: Regression diagnostics and influential data. In: R.R. Watson (ed.), Drug and Alcohol Abuse Reviews: Drug and Alcohol Abuse Prevention (pp. 223-43). Clifton, NJ: Human Press.

108. Nelson, J.P. (1997). Economic and demographic factors in U.S. alcohol demand: A growth-accounting analysis. Empirical Economics, 22, 83-102.

109. Nelson, J.P. (1999). Broadcast advertising and U.S. demand for alcoholic beverages. Southern Economic Journal, 65, 774-90.

110. Nelson, J.P. (2003). Advertising bans, monopoly, and alcohol demand: Testing for substitution effects using state panel data. Review of Industrial Organization, 22, 1-25.

111. *Nelson, J.P. (2010). Alcohol advertising bans, consumption and control policies in seventeen OECD countries, 1975-2000. Applied Economics, 42, 803-23.

112. Nelson, J.P. and Moran, J.R. (1995). Advertising and US alcoholic beverage demand: System-wide estimates. Applied Economics, 27, 1225-36.

113. Nelson, J.P. and Young, D.J. (2001). Do advertising bans work? An international comparison. International Journal of Advertising, 20, 273-96.

114. Niskanen, W.A. (1962). The demand for alcoholic beverages, Rand Research Report P-2583. Santa Monica: Rand Corporation.

115. Norman, D.A. (1976). Structural Change and Performance in the U.S. Brewing Industry. Unpublished Ph.D. dissertation, Department of Economics, University of California, Los Angeles.

116. Norstrom, T. (2005). The price elasticity for alcohol in Sweden 1984-2003. Nordic Studies on Alcohol and Drugs, 22, English Supplement, 87-101.

117. *Ogwang, T. and Cho, D.I. (2009). Economic determinants of the consumption of alcoholic beverages in Canada: A panel data analysis. Empirical Economics, 37, 599-613.

118. Ornstein, S.I. and Hanssens, D.M. (1985). Alcohol control laws and the consumption of distilled spirits and beer. Journal of Consumer Research, 12, 200-13.

119. *Osoro, N.E., Mwinyimvua, H.H., and Mpango, P.I.N. (2005). Performance and revenue potential of excise taxation in Tanzania. Journal of African Economics, 15, 1-25.

120. Owen, A.D. (1979). The demand for wine in Australia, 1955-1977. Economic Record, 55, 230-35.

121. *Ozguven, C. (2004). Analysis of Demand and Pricing Policies in Turkey Beer Market. Unpublished Masters Thesis, Middle East Technical University.

122. Pagoulatos, E. and Sorensen, R. (1986). What determines the elasticity of industry demand? International Journal of Industrial Organization, 4, 237-50.

123. *Pan, S., Fang, C. and Malaga, J. (2006). Alcoholic beverage consumption in China: A censored demand system approach. Applied Economics Letters, 13, 975-79.

**178**. Partenan, J. (1991). Sociability and intoxication: Alcohol and drinking in Kenya, Africa and the modern world. Helsinki: Finnish Foundation for Alcohol Studies. **OUT-OF-ORDER**

124. Pearce, D. (1985). The demand for alcohol in New Zealand. Working paper, Department of Economics, University of Western Australia.

125. Penm, J. (1988). An econometric study of the demand for bottled, canned and bulk beer. Economic Record, 64, 268-74.

**126-127**. *Pierani, P. and Tiezzi, S. (2007). Addiction and alcohol consumption: Evidence from Italian data. Rivista Internazionale di Science Sociali, 115, 265-84.

128. *Pierani, P. and Tiezzi, S. (2009). Addiction and interaction between alcohol and tobacco consumption. Empirical Economics, 37, 1-23.

129. *Pierani, P. and Tiezzi, S. (2011). Infrequency of purchase, individual heterogeneity and rational addiction in single households’ estimates of alcohol consumption, Giornale degli Economisti e annali de Economia, 70, 93-116.

130. Quek, K.E. (1988). The demand for alcohol in Canada: An econometric study. Working paper, Department of Economics, University of Western Australia.

131. *Ruhm, C.J., et al. (2011). What U.S. data should be used to measure the price elasticity of demand for alcohol? NBER Working paper 17578, National Bureau of Economic Research.

132. Sabuhoro, J.B., Laru, B., and Lariviere, E. (1996). Advertising expenditures and the consumption of alcoholic beverages. Journal of International Food & Agribusiness Marketing, 8, 37-54.

133. Saffer, H. and Dave, D. (2002). Alcohol consumption and alcohol advertising bans. Applied Economics, 34, 1325-34.

134. Salisu, M.A. and Balasubramanyam, V.N. (1997). Income and price elasticities of demand for alcoholic drinks. Applied Economics Letters, 4, 247-51.

135. *Sam, A. and Thompson, S. (2012). Impact of country of origin advertising on U.S. import wine demand: A panel data analysis. Applied Economics Letters, 19, 1871-77.

136. Schweitzer, S.O., Intrilligator, M.D., and Salehi, H. (1983). Alcoholism: An econometric model of its causes, its effects and its control. In: M. Grant, M. Plant, and A. Williams (eds.), Economics and alcohol: Consumption and controls (pp. 107-27). New York: Gardner Press.

137. *Seale, J.L., Marchant, M.A. and Basso, A. (2003). Imports versus domestic production: A demand system analysis of the U.S. red wine market. Review of Agriculture Economics, 25, 187-202.

138. Selvanathan, E.A. (1988). Alcohol consumption in the UK, 1955-85: A system-wide analysis. Applied Economics, 20, 1071-86.

139. Selvanathan, E.A (1989). Advertising and alcohol demand in the UK: Further results. International Journal of Advertising, 8, 181-88.

140. Selvanathan, E.A (1991). Cross-country alcohol consumption comparison: An application of the Rotterdam demand system. Applied Economics, 23, 1613-22.

141. Selvanathan, E.A (1995). The effects of advertising on alcohol consumption: An empirical analysis. In: E.A. Selvanathan and K.W. Clements (eds.), Recent Developments in Applied Demand Analysis: Alcohol, Advertising and Global Consumption (pp. 297-340). Berlin: Springer.

142. Selvanathan, E.A and Selvanathan, S. (2004). Economic and demographic factors in Australian alcohol demand. Applied Economics, 36, 2405-17.

143. *Selvanathan, S. and Selvanathan, E.A. (2005a). Empirical regularities in cross-country alcohol consumption. Economic Record, 81, S128-42.

**144-145**. Selvanathan, S. and Selvanathan, E.A. (2005b). Demand for beer, wine and spirits. In: The demand for alcohol, tobacco and marijuana: International evidence (pp. 211-41). Aldershot, UK: Ashgate.

146. *Selvanathan, S. (2006). How similar are alcohol drinkers? International evidence. Applied Economics, 38, 1353-62.

147. Selvanathan, S. and Selvanathan, E.A. (2007). Another look at the identical tastes hypothesis on the analysis of cross-country alcohol data. Empirical Economics, 32, 185-215.

148. *Shi, Y. (2011). Three Essays on Economics of Health Behavior in China. Unpublished Ph.D. dissertation, Pardee RAND Graduate School.

149. Smith, R.T. (1976). The legal and illegal markets for taxed goods: Pure theory and an application to state government taxation of distilled spirits. Journal of Law and Economics, 19, 393-429.

150. Stone, R. and Rowe, D.A. (1958). Dynamic demand functions: Some econometric results. Economic Journal, 68, 256-70.

151. Swidler, S. (1986). A rexamination of liquor price and consumption differences between public and private ownership states: Comment. Southern Economic Journal, 53, 259-68.

152. *Taube, P.M., Huth, W.L., and MacDonald, D.N. (1990). An analysis of consumer expectation effects on demand in a dynamic almost ideal demand system. Journal of Economics and Business, 42, 225-36.

153. *Taube, P.M. and MacDonald, D.N. (1991). A dynamic almost ideal demand system incorporating consumer expectations. Managerial and Decision Economics, 12, 197-206.

154. Tegene, A. (1990). The Kalman filter approach for testing structural change in the demand for alcoholic beverages in the US. Applied Economics, 22, 1407-16.

155. Thom, D.R. (1984). The demand for alcohol in Ireland. Economic and Social Review, 15, 325-36.

156. *Tian, G. and Liu, F. (2011). Is the demand for alcoholic beverages in developing countries sensitive to price? Evidence from China. International Journal of Environmental Research and Public Health, 8, 2124-31.

**179**. *Tiffin, R. et al. (2011). Estimating food and drink elasticities, University of Reading Working Paper, Report to UK Defra. **OUT-OF-ORDER**.

157. *Treisman, D. (2010). Death and prices: The political economy of Russia’s alcohol problem. Economics of Transition, 18, 281-331.

158. Trolldal, B. and Ponicki, W. (2005). Alcohol price elasticities in control and license states in the United States, 1982-99. Addiction, 100, 1158-65.

**180.** *Trancoso-Valverde, C. (2004). Structural breaks, cointegration and the domestic demand for Chilean wine, Working paper, University of Talca. **OUT-OF-ORDER**.

159. Uri, N.D. (1986). The demand for beverages and interbeverage substitution in the United States. Bulletin of Economic Research, 38, 77-85.

160. *Volland, B. (2009). Elasticities of German beer demand revisited. Working paper, Max Planck Institute of Economics.

161. Walsh, B.M. (1982). The demand for alcohol in the UK: A comment. Journal of Industrial Economics, 30, 439-46.

162. Walsh, B.M. and Walsh, D. (1970). Economic aspects of alcohol consumption in the Republic of Ireland. Economic and Social Review, 2, 115-38.

163. Wang, J., Gao, X.M., Wailes, E.J., and Cramer, G.L. (1996). U.S. consumer demand for alcoholic beverages: Cross-section estimation of demographics and economic effects. Review of Agricultural Economics, 18, 477-89.

164. Wang, Q., Halbrendt, C.C., and Jensen, H.H. (1997). China’s beer consumption and barley imports. Agribusiness, 13, 73-84.

165. *West, S.E. and Parry, I.W.H. (2009). Alcohol-leisure complementarity: Empirical estimates and implications for tax policy. National Tax Journal, 62, 611-33.

166. Wette, H.C., Zhang, J-F., Berg, R.J., and Casswell, S. (1993). The effect of prices on alcohol consumption in New Zealand 1983-1991. Drug and Alcohol Review, 12, 151-58.

167. Wilkinson, J.T. (1987a). Reducing drunken driving: Which policies are most effective? Southern Economic Journal, 54, 322-34.

168. *Wilkinson, J.T. (1987b). The effects of regulation on the demand for alcohol. Working paper, Department of Economics, University of Missouri.

169. *Wohlgenant, M.K. (2011). Rational addiction in the U.S. demand for wine. Working paper, Monash University and North Carolina State University.

170. Yen, S.T. (1994). Cross-section estimation of US demand for alcoholic beverage. Applied Economics, 26, 381-92.

171. *Yu, X. and Abler, D. (2010). Interactions between cigarette and alcohol consumption in rural China. European Journal of Health Economics, 11, 151-60.

172. Yu, W. and Chen, L. (1998). The demand for alcoholic beverages in New Brunswick, Canada: A cointegration analysis. Canadian Journal of Regional Science, 21, 1-14.

173. *Zereyesus, Y.A. (2010). Essays in Applied Demand and Production Analysis. Unpublished Ph.D. dissertation, Department of Agricultural Economics, Kansas State University.

174. Zhang, J-F. and Casswell, S. (1999). The effects of real price and a change in the distribution system on alcohol consumption. Drug and Alcohol Review, 18, 371-78.

175. *Zhuk, O. (2011). Advertising, drinking age laws, and the demand for beer. Working paper, Economics Program, University of Texas at Dallas.

176. *Zoltan, G. (2006). Transformation of the Hungarian beer market in the light of international tendencies. Unpublished Ph.D. dissertation, Corvinus University of Budapest.

**IIB. GALLET & FOGARTY STUDIES EXCLUDED** [reason for omission]

**Ahtola, J., Ekhom, A., and Somervuori, A. (1986). Bayes estimates for the price and income elasticities of alcoholic beverages in Finland, 1955-1980. Journal of Business and Economic Statistics, 4, 199-208. [no std. errors]

**Atkinson, A.B., Gomulka, J., and Stern, N.H. (1990). Spending on alcohol: Evidence from the Family Expenditure Survey, 1970-1983. Economic Journal, 100, 808-27. [older data]

**Baker, P. and McKay, S. (1990). The structure of alcohol taxes: A hangover from the past? London: Institute for Fiscal Studies. [no std errors or t-statistics reported]

**Bentzen, J., Eriksson, T., and Smith, V. (1999). Rational addiction and alcohol consumption: Evidence from the Nordic Countries. Journal of Consumer Policy, 22, 257-79. [no std. errors]

**Blaylock, J.R. and Blisard, W.N. (1993). Women and the demand for alcohol: Estimating participation and consumption. Journal of Consumer Affairs, 27, 319-34. [income only, no prices]

**Blaylock, J.R. and Blisard, W.N. (1993). Wine consumption by US men. Applied Economics, 24, 645-51. [income only, no prices]

**Bourgeois, J.C. and Barnes, J.G. (1979). Does advertising increase alcohol consumption? Journal of Advertising Research, 19, 19-29. [relative beverage prices]

**Buccola, S.T. and VanderZanden, L. (1997). Wine demand, price strategy, and tax policy. Review of Agricultural Economics, 19, 428-40. [wine by color varietal, Oregon vs. CA/WA]

**Butter, F.A.C., Delifotis, A., and Koning, R.H. (1997). Preference shifts in customer demand for beer and wine. Working paper, Free University: Amsterdam. [relative beverage prices]

**Clements, K.W. and Selvanathan, S. (1995). The demand for alcoholic beverages. In: E.A. Selvanathan and K.W. Clements (eds.), Recent developments in applied demand analysis: Alcohol, advertising and global competition (pp. 221-57. Berlin: Springer. [same at 1991 paper]

**Conrad, K. (1989). Tests for optimizing behavior and for patterns of conjectural variations. Kyklos, 42, 231-55. [brand data, one company]

**Cook, P.J. and Tauchen, G. (1982). The effect of liquor taxes on heavy drinking. Bell Journal of Economics, 13, 379-90. [taxation study]

** Crawford, I. and Tanner, S. (1995b). Alcohol taxes and the single European market. Unpublished working paper, Institute for Fiscal Studies. [same value as 1995a paper, duplicate]

**Duffy, M. (1981). The influence of prices, consumer incomes and advertising upon the demand for alcoholic drink in the United Kingdom: An econometric study. British Journal on Alcohol and Alcoholism, 16, 200-208. [no std errors or t-statistics reported]

**Folwell, R.J. and Baritelle, J.L. (1978). The U.S. wine market. Washington, DC: U.S. Department of Agriculture. [wine by varietal and census region]

**Franke, G. and Wilcox, G. (1987). Alcoholic beverage advertising and consumption in the United States, 1964-1984. Journal of Advertising, 16, 22-30. [no prices]

**Freeman, D.G. (1999). A note on ‘economic conditions’ and alcohol problems. Journal of Health Economics, 18, 661-70. [beer tax rates]

**Freeman, D.G. (2000). Alternative panel estimates of alcohol demand, taxation, and the business cycle. Southern Economic Journal, 67, 325-44. [beer tax rates]

**Freeman, D.G. (2011). Cold comfort in hard times: Do people drink more beer during recessions? in J.F.M. Swinnen (ed.), The Economics of Beer. Oxford: Oxford University Press, pp. 107-22. [no elasticities]

**Gius, M.P. (1996). Using panel data to determine the effect of advertising on brand-level distilled spirits sales, Journal of Studies on Alcohol, 57, 73-76. [brand data]

**Grabowski, H.G (1976). The effects of advertising on the interindustry shifts of demand. Explorations in Economic Research, 4, 675-701. [firm data]

**Grossman, M., Chaloupka, F.J., and Sirtalan, I. (1998). An empirical analysis of alcohol addiction: Results from the Monitoring the Future panels. Economic Inquiry, 36, 39-48. [taxation study, survey data]

**Grossman, M. and Markowitz, S. (2005). Alcohol regulation and violence on college campuses. In: B. Lindgren and M. Grossman (eds.), Substance use: Individual behaviour, social interactions, markets and politics, vol. 16 (pp. 257-89). Amsterdam: Elsevier. [taxation study, survey data]

**Hausman, J., Leonard, G., and Zona, J.D. (1994). Competitive analysis with differentiated products. Annales d’Economie et de Statistique, 17, 159-80. [brand/firm level data]

**Kenkel, D.S. (1996). New estimates of the optimal tax on alcohol. Economic Inquiry, 34, 296­-319. [survey data]

**Larue, B., Ker, A., and MacKinnon, L. (1991). The demand for wine in Ontario and the phasing-out of discriminatory mark-ups. Agribusiness, 7, 475-88. [varietal study; imports by country]

**Levy, D. and Sheflin, N (1985). The demand for alcoholic beverages: An aggregate time-series analysis. Journal of Public Policy and Marketing, 4, 47-54. [duplicate, same as 1983 results]

**Lynk, W.J. (1984). Interpreting rising concentration: The case of beer. Journal of Business, 57, 43-55. [income only, no prices]

**Malmquist, S. (1948). A statistical analysis of the demand for liquor in Sweden. Working paper, University of Uppsala. [older data]

**Mast, B.D, Benson, B.L., and Rasmussen, D.W. (1999). Beer taxation and alcohol-related traffic fatalities. Southern Economic Journal, 66, 214-49. [taxation study]

**Mayo, J. (2000). An estimate of U.S. demand for alcoholic beverages, 1986-92. Pennsylvania Economic Review, 9, 1-4. [poor quality, std. errors uncertain]

**McGahan, A.M. (1995). Cooperation in prices and capacities: Trade associations in brewing after Repeal. Journal of Law and Economics, 38, 521-57. [taxation study]

**Miller, G.L. and Roberts, I.M. (1972). The effect of price change on wine sales in Australia. Quarterly Review of Agricultural Economics, 25, 231-39. [arc elasticity, no std. errors]

**Musgrave, S. and Stern, N. (1988). Alcohol: Demand and taxation under monopoly and oligopoly in South India in the 1970s. Journal of Development Economics, 28, 1-41. [arrack/homebrew study, no beer results]

**Nayga, R.M. and Capps, O. (1994). Analysis of alcohol consumption in the United States: Probability and level of intake. Journal of Food Distribution Research, 25, 17-23. [income only, no prices, survey data]

**Nerlove, M. and Addison, W. (1958). Statistical estimation of long-run elasticities of supply and demand. Journal of Farm Economics, 40, 861-80. [older data]

**Pacula, R.L. (1998). Does increasing the beer tax reduce marijuana consumption? Journal of Health Economics, 17, 557-85. [taxation study, survey data]

**Pompelli, G. and Heien, D. (1991). Discrete/continuous consumer demand choices: An application to the U.S. domestic and imported white wine markets. European Review of Agricultural Economics, 18, 117-30. [survey data]

**Prest, A.R. (1949). Some experiments in demand analysis. Review of Economics and Statistics, 31, 33-49. [older data]

**Saffer, H. (1991). Alcohol consumption and alcohol advertising bans. Journal of Health Economics, 10, 65-79. [semilog model, elasticities not reported]

**Sass, T.R. and Saurman, D. S. (1993). Mandated exclusive territories and economic efficiency: An empirical analysis of the malt-beverage industry. Journal of Law and Economics, 36, 153­-77. [taxation study, total sales, linear model]

**Sass, T.R. and Saurman, D.S. (2001). Mandated exclusive territories: Efficiency effects and regulatory selection bias. In: M.R. Baye and J.P. Nelson (eds.), Advances in Applied Microeconomics: Advertising and Differentiated Products, Vol. 10 (pp. 55-72). Amsterdam: JAI Press. [taxation study]

**Simon, J.L. (1966). The price elasticity of liquor in the U.S. and a simple method of determination. Econometrica, 34, 193-205. [experimental study, std. errors not possible]

**Skog, O-L. and Melberg, O. (2006). Becker’s rational addiction theory: An empirical test with price elasticities for distilled spirits in Denmark 1911-31. Addiction, 101, 1444-50. [older data]

**Spurry, S. (1999). Demographic influence on the U.S. demand for beer. Issues in Political Economy, 8, 1-12. [brand data, linear model]

**Stone, R. (1945). The analysis of market demand. Journal of the Royal Statistical Society, 108, 1-98. [older data].

**Stone, R. (1951). The role of measurement in economics. Cambridge: Cambridge University Press.[older data].

**Sander, W. (1999). Cognitive ability, schooling and the demand for alcohol by young adults. Education Economics, 7, 53-66. [income only, no prices]

**Su, S-J. and Yen, S.T. (2000). A censored system of cigarette and alcohol consumption. Applied Economics, 32, 729-37. [income only, no prices]

**Taplin, J. and Ryan, W. 1969). The prospects for wine in Australia, Quarterly Review of Agricultural Economics, 22, 198-202. [arc elasticity]

**Tsolakis, D. (1983), Taxation and consumption of wine, Review of Marketing and Agricultural Economics, 51, 155-65. [no demand]

**Tsolakis, D., Riethmuller, P., and Watts, G. (1983). The demand for wine and beer. Review of Marketing and Agricultural Economics, 51, 131-53. [relative beverage price model]

**Wales, T.J. (1968). Distilled spirits and interstate consumption effects. American Economic Review, 58, 853-63. [no std. errors for elasticities]

**Wong, A.T-T. (1988). The demand for alcohol in the UK 1920-1938: An econometric study. Working paper No. 88-13. Department of Economics,. University of Western Australia. [older data]

**Yamada, T., Kendrix, M., and Yamada, T. (1993). The impact of alcohol consumption and marijuana use on high school graduation. Health Economics, 5, 77-92. [taxation study]

**Yen, S.T. (1995). Alternative transformations in a class of limited dependent variable models: Alcohol consumption by US women. Applied Economics Letters, 258-62. [income only, no prices]

**Yen, S.T. and Jensen, H.H. (1996). Determinants of household expenditures on alcohol. Journal of Consumer Affairs, 30, 48-67. [income only, no prices]

**Young, D.J. and Bielinska-Kwapisz, A. (2001). Alcohol consumption, beverage prices and measurement error. Journal of Studies on Alcohol, 64, 235-38. [taxation study]

**IC. OTHER EXCLUDED STUDIES FROM META-ANALYSIS** [reason]

**Abdel-Ghany, M. and Silver, J.L. (1998). Economic and demographic determinants of Canadian households’ use of and spending on alcohol. Family and Consumer Sciences Research Journal, 27, 62-90. [income only]

**Acheson, K. (1977). Revenue vs protection: The pricing of wine by the Liquor Control Board. Canadian Journal of Economics, 10, 246-62. [linear demand, no elasticities reported]

**Adrian, M. and Ferguson, B.S. (1986). The influence of income on the consumption of alcohol in Ontario: A cross-section study. In A. Carmi and S. Schneider (eds.), Drugs and Alcohol. Berlin: Springer-Verlag, pp. 151-57. [income only]

**Alcordo, E.A. and Johnson, L.W. (1985). An alternative approach to the specification of approximate demand systems. Australian Economic Papers, 24, 380-93. [duplicate]

**Arnoult, M. and Tiffin, R. (2010), Minimum pricing of alcohol and its impact on consumption in the UK. Unpublished paper, Scottish Agricultural College and University of Reading. [no std. errors reported]

**Beard, T.R., Gant, P.A., and Saba, R.P. (1997). Border-crossing sales, tax avoidance, and state tax policies: An application to alcohol. Southern Economic Journal, 64, 293-306 [tax effects]

**Beelitz, F. (2009). Evaluating coordinated effects: An application to the US beer industry, Research Report Series No. 114, University of Connecticut. [brand data]

**Benson, B.L., Rasmussen, D.W., and Zimmerman, P.R. (2003) Implicit taxes collected by state liquor monopolies. Public Choice, 115, 313-31. [tax study]

**Bentzen, J. and Smith, V. (2005), Short-run and long-run relationships in the consumption of alcohol in the Scandinavian countries, Danish Economic Journal *(*(Nationaløkonomisk Tidsskrift)*,* 143, 65-80*.* [no elasticities]

**Berggren, F. and Sutton, M. (1999). Are frequency and intensity of participation decision-bearing aspects of consumption? An analysis of drinking behavior. Applied Economics, 31, 865-74. [no prices]

**Bettocchi, A. and Mazzocchi, M. (2003). Demand for alcoholic beverages in Italy and socio-demographic factors. In S. Gatti et al. (eds.), Wine in the Old World: New Risks and Opportunities. Milano: FrankoAngeli, pp. 129-42. [no std errors]

**Browning, M. (1987). Eating, drinking, smoking, and testing the lifecycle hypothesis. Quarterly Journal of Economics, 92, 329-45. [no std errors]

**Burkey, M.L. (2010) Geographic access and demand in the market for alcohol. Review of Regional Studies, 40, 159-79. [income only]

**Busch, S.H. et al. (2004). Burning a hole in the budget: Tobacco spending and its crowd-out of other goods, Applied Health Economics, 3, 263-72. [different model, elasticities for alcohol are outliers]

**Carew, R.. Florkowski, W.J., and He, S. (2004). Demand for domestic and imported table wine in British `Columbia: A source-differentiated almost ideal demand system approach. Canadian Journal of Agricultural Economics, 52, 183-99. [varietal data]

**Castiglione, C., Grochova, L., Infante, D., and Smirnova, J. (2011). The demand for beer in the presence of past consumption and advertising in the Czech Republic. Agricultural Economics–Czech, 57, 589-99. [brands}

**Chaiyasong, S., et al. (n.d.). Alcohol consumptioin after the spirits tax increase in Thailand: Results from a community-based survey and an estimated price increase. Khon Kaen University. [elasticity source in Thai]

**Cho, D.I. and Permyakov, M. (2007). Structural changes in the demand for wine in Canada. International Journal of Wine Business Research, 19, 311-26. [year-to-year elasticities]

**Collis, J., Czubek, M., and Johal. S. (2010). Estimating price elasticities of demand for alcohol and tobacco in the UK, Working paper. London: HM Revenue & Customs. (duplicate).

**Cuellar, S.S., Colgan, T., Hunnicutt, H., and Ransom, G. (2010). The demand for wine in the USA, International Journal of Wine Business Research, 22, 178-90. [varietal data]

**Cuellar, S.S. and Huffman, R. (2008). Estimating the demand for wine using instrumental variable techniques, Journal of Wine Economics, 3, 1-13. [varietal data]

**David, T., Ahmadi-Esfahani, A., and Iranzo, S. (2007). Demand under product differentiation: An empirical analysis of the US wine market. Unpublished paper, University of Sydney. [varietal data]

**Davis, T., Ahmadi-Esfahani, A., and Iranzo, S. (2007). Demand under product differentiation: An empirical analysis of the US wine market, Working paper, University of Sydney. [varietal data]

**Deaton, A. and Irish, M. (1984) Statistical models for zero expenditures in household budgets. Journal of Public Economics, 23, 59-80. [no prices]

**Dong, Y. (2011). Semiparametric binary random effects models: Estimating two types of drinking behavior. Economics Letters, 112, 79-81. [no prices]

**Fan, S., Wailes, E.J. and Cramer, G.L. (1995). Household demand in rural China: A two-stage LES_AIDS model, American Journal of Agriucultural Economics, 77, 54-62. [no std. errors]

**Faroque, A. (2007). An investigation into the demand for alcoholic beverages in Canada: A choice between the almost ideal and Rotterdam models. Applied Economics, 40, 2045-54. [no elasticities reported]

**Franses, P.H. (1991). Primary demand for beer in The Netherlands: An application of the ARMAX model specification, Journal of Marketing Research, 28, 240-45. [no elasticities reported]

**Fauntleroy, P.A. (1984). An Economic Analysis of the United States Demand for Distilled Spirits, Wine, and Beer Incorporating Taste Changes Through Demographic Factors, 1960-1981. Unpublished Ph.D. dissertation, American University. [brand data]

**Fogarty, J.J. (2006). The nature of the demand for alcohol: Understanding elasticity. British Food Journal, 106, 316-31. [literature review]

**Fuller, K.B. and Alston, J.M. (2012). The demand for winegrapes in California. Unpublished paper, University of California, Davis. [varietal data]

**Gardes, F. and Starzec, C. (2002). Evidence on addiction from household expenditure surveys: The case of Polish consumers. Unpublished paper, Universite Paris and INSEE. [no elasticities reported]

**Gardes, F. and Starzec, C. (2004). Are tobacco and alcohol expenditures price elastic? The case of Poland consumption. Unpublished paper, Universite Paris and INSEE. [no std errors]

**Gerolimetta, M., Mauracher, C., and Procidano, I. (2008). Analyzing wine demand with artificial neural networks. Journal of Wine Economics, 3, 30-50. [no std. errors]

**Gil, A.I. and Molina, J.A. (2009) Alcohol demand among young people in Spain: An addictive QUAIDS. Empirical Economics, 36, 515-30. [ages 14-18 years]

**Ground, M. and Koch, S.F. (2008). Hurdle models of alcohol and tobacco expenditure in South African households. South African Journal of Economics, 76, 132-43. [no prices]

**Ground, M., Koch, S., and van Wyk, D. (2008). South African household expenditure patterns: Alcohol products in 1995 and 2000, Acta Academica, 40, 127-60. [no prices]

**Harris, D.J. (1964) Econometric analysis of household consumption in Jamaica. Social and Economic Studies, 13, 471-87. [no prices]

**Haque, M.Q. (1990). The demand for alcohol in Australia. Drug and Alcohol Review, 9, 43-52. [no prices]

**Heck, T. and Kochanowski, P. (1986). Per capita alcohol consumption in the U.S.: Some empirical findings. Journal of the Southwestern Society of Economists, 13, 78-85. [no elasticities reported]

**Heck, T. and Kochanowski, P. (1986) The effects of government policy on alcohol consumption. Proceedings of the Seventeenth Annual Pittsburgh Conference on Modeling and Simulation, pp. 345-52. [no elasticities]

**Hoadley, J.F., Fuchs, B.C., and Holder, H.D. (1984). The effects of alcohol beverage restrictions on consumption: A 25-year longitudinal analysis. American Journal of Drug and Alcohol Abuse, 10, 375-401. [no elasticities]

**Horowitz, I. and Horowitz, A.R. (1965). Firms in a declining market: The brewing case. Journal of Industrial Economics, 13, 129-53. [tax study]

**Johnson, J.A., Oksanen, E.H., Veall, M.R., and Fretz, D. (1990). Alternative approaches to the measurement of consumption and price of alcoholic beverages, Canada, 1957-1983. Journal of Studies on Alcohol, 51, 82-85. [no elasticities]

**Kacapyr, E. and Choudhury, S. (2006). Determinants of alcohol consumption by college students. New York Economic Review, 37, 3-19. [no prices]

**Karingi, S.N., Kimenyi, M.S., and Ndung’u, N.S. (2001) Beer taxation in Kenya: An assessment. KIPPRA Discussion Paper No. 6, Kenya Institute for Public Policy Research and Analysis, Nairobi. [brand data]

**Kennedy, K.A., Walsh, B.M., and Ebrill, L.P. (1973). The demand for beer and spirits in Ireland. Proceeding of the Royal Irish Academy, 73, 669-711. [linear models]

**Kioulafas, K.E. (1985). An application of multiple regression analysis to the Greek beer market. Journal of the Operations Research Society, 36, 689-96. [firm data]

**Kitchin, P.D. Socio-economic determinants of UK alcohol consumption, 1956-79. International Journal of Social Economics, 10, 34-9. [no elasticities]

**Koksalan, M., Erkip, N., and Moskowitz, H. (1999). Explaining beer demand: A residual modeling regression approach. International Journal of Production Economics, 58, 265-76. [brand data]

**Koksal, A. and Wohlgenant, M. (2010). Alcoholic beverages and cigarettes: Complement or substitutes? A pseudo panel approach, Working paper, North Carolina State University. [duplicate, used Koksal (2012)]

**Koksal, A. and Wohlgenant, M. (2011). Rationally addicted to cigarettes, alcohol and coffee? A pseudo panel approach, Working paper, North Carolina State University. [duplicate, used Koksal (2012)]

**Kubik, J.D. and Moran, J.R. (2001). Can policy changes be treated as natural experiments” Evidence from state excise taxes. Unpublished paper, Syracuse University. [tax study]

**Kusuda, Y. (2011). Nested logit demand estimation in Japanese beer-like beverage markets, Working paper, Nohon Fukushi University. [brand data]

**La Cour, L. and Milhoj, A. (2009). The sale of alcohol in Denmark – Recent development and dependencies on prices/taxes, Applied Economics, 41, 1089-103. [no elasticities]

**Langan, G.E. (1997). Brand level demand and oligopolistic price interaction among domestic and foreign beer brands. Unpublished Ph.D. dissertation, University of Connecticut. [brand data]

**Lee, Y., Kennedy, P.L., and Hilbun, B.M. (2009). A demand analysis of the Korean wine market using an unrestricted source differentiated LA/AIDS model. Journal of Wine Economics, 4, 185-200. [varietal data]

**Leeflang, P.S.H. and van Duijn, J.J. (1982) The use of regional data in marketing models: The demand for beer in the Netherlands. European Research, 10, 2-9 and 64-71. [linear models]

**Lopez, R.A. and Matschke, X. (2007). Home bias in U.S. beer consumption. Unpublished paper, University of Connecticut. [brand data]

**Luksetich, W.A. (1975). A study of regulation: The Minnesota liquor case. Southern Economic Journal, 41, 457-65. [brand data]

**Madden, D. (2008). Sample selection versus two-part models revisited: The case of female smoking and drinking. Journal of Health Economics, 27, 300-07. [no prices]

**Mahal, A. (2000). What works in alcohol policy? Evidence from rural India. Economic and Political Weekly, 35, 3959-68. [no elasticities]

**Manrique, J. and Jensen, H.H. (2004). Consumption of tobacco and alcoholic beverages among Spanish consumers. Southwestern Economic Review, 31, 41-56. [no prices]

**Matthews, K., et al. (2006). Violence-related injury and the price of beer in England and Wales. Applied Economics, 38, 661-70. [no demand analysis}

**Mayo, J.R. (2000). An estimate of U.S. demand for alcoholic beverages, 1986-92. Pennsylvania Economic Review, 9, 1-4. [very poor model]

**Maynard, A. (1988) Price as a determinant of alcohol consumption. Australian Drug and Alcohol Review, 7, 287-96. [review study]

**Mazzocchi, M. (2006). Time patterns in UK demand for alcohol and tobacco: An application of the EM algorithm. Computational Statistics & Data Analysis, 50, 2191-205. [no elasticities]

**Meloche, M.S. and Stanton, J.L. (2009). A comparison of distilled spirit consumption patterns in the United Kingdom and the United States: A syndicated data approach. Worldwide Hospitality and Tourism Themes, 1, 162-68. [no prices]

**McCullough, M.P., Smith, T.G., and Marsh, T.L. (2012). Intertemporal dependence in alcohol consumption data: Evidence of rational addiction? Ecometrica, 5, 1-25. [no prices]

**Miravete, E.J., Thurk, J., Seim, K. and Waldfogel, J. (2012). Complexity, efficiency, and fairness in multiproduct liquor pricing. Unpublished working paper, University of Texas. [brand data]

**Muhammad, A. (2011). Wine demand in the United Kingdom and New World structural change: A source-disaggregated analysis, Agribusiness, 27, 82-98. [imports by country]

**Nayga, R.M. and Capps, O. (1994) Analysis of alcohol consumption in the United States: Probability and level of intake. Journal of Food Distribution Research, 25, 17-23. [no prices]

**Nayga, R.M. (1996). Sample selectivity models for away from home expenditures on wine and beer. Applied Economics, 28, 1421-25. [no prices]

**Nelson, J.P. (2010). Alcohol, unemployment rates and advertising bans: International panel evidence, 1975-2000. Journal of Public Affairs, 10, 74-87. [duplicate study]

**Nicita, A. (2008). Price elasticities and tax reform in Mexico, Applied Economics, 40, 2329-35. [no separate results for alcohol]

**Okello, A.K. (2001) An analysis of excise taxation in Kenya. African Economic Policy Discussion Paper No. 73, John F. Kennedy School of Government, Harvard University, [brand data]

**Okrent, A.M. and Alston, J.M. (2012). The demand for disaggregated food-away-from-home and food-at-home products in the United States, Working paper, Economic Research Service, USDA, and University of California, Davis. [alcohol consumption data uncertain]

**O’Riordan, W.K. (1975). An application of the Rotterdam demand system to Irish data, Economic and Social Review, 6, 511-29. [no separate results for alcohol]

**O’Riordan, W.K. (1976). Consumer response to price and income changes, Journal of the Statistical and Social Inquiry Society of Ireland, 23, 65-84. [no std. errors]

**Pappas, L.S. (1986). Optimal taxation of alcohol and tobacco in the presence of externalities: An econometric analysis. Unpublished Ph.D. dissertation, SUNY at Binghampton. [NIPA data]

**Paulin, G. (2003). Consumer expenditures for alcohol in 2000. Consumer Expenditure Survey Anthology, 2003, 39-59. [no prices]

**Peltzman, S. (1971). Pricing in public and private enterprises: Electric utilities in the United States. Journal of Law and Economics, 14, 109-47. [brand data]

**Ponicki, W. et al. (1997). Altering alcohol price by ethanol content: Results from a Swedish tax policy in 1992, Addiction, 92, 859-70. [brand data]

**Rabinovich, L. et al. (2009). The affordability of alcoholic beverages in the European Union: Understanding the links between alcohol affordability, consumption and harms. Rand Europe. [no standard errors, p. 44]

**Ramful, P. and Zhao, X. (2008). Individual heterogeneity in alcohol consumption: The case of beer, wine and spirits in Australia. Economic Record, 84, 207-22. [participation elasticities]

**Rice, N. and Sutton, M. (1998). Drinking patterns within households: The estimation and interpretation of individual and group variables. Health Economics, 7, 689-99. [no prices]

**Rojas, C. (2008). Price competition in U.S. brewing. Journal of Industrial Economics, 56, 1-31. [brand data]

**Rojas, C. and Peterson, E.V. (2008). Demand for differentiated products: Price and advertising evidence from the U.S. beer market. International Journal of Industrial Organization, 26, 288-307. [brand data]

**Rojas, C. and Shi, T. (2011). Tax incidence when quality matters: Evidence from the beer market. Journal of Agricultural & Food Industrial Organization, 9, article 10. [brand data]

**Sharpe, D.L., Abdel-Ghany, M., Kim, H-Y., and Hong, G-S. (2001). Alcohol consumption decisions in Korea. Journal of Family and Economic Issues, 22, 7-24. [no prices]

**Shapouri, S., Folwell, R.J., and Baritelle, J.L. (1981). Statistical estimation of firm-level demand functions: A case study in an oligopolistic industry. Agricultural Economics Research, 33, 18-25. [firm varietal data]

**Smith, D.I. (1990). Consumption and advertising of alcoholic beverages in Australia, 1969-86. Drug and Alcohol Review, 9, 33-43. [no prices]

**Smith, J.T. (1982). An analysis of state regulations governing liquor store licenses. Journal of Law and Economics, 25, 301-19.[no demand estimates]

**Stehr, Mark . (2007). The effect of Sunday sales bans and excise taxes on drinking and cross border shopping for alcoholic beverages, National Tax Journal, 60, 85-105. [smuggling model; tax elasticites]

**Stockwell, T. et al. (2011). Does minimum pricing reduce alcohol consumption? The experience of a Canadian province. Addiciton, 107, 913-920. [elasticities based on minimum price changes].

**Stockwell, T., et al. (2012). The raising of minimum alcohol prices in Saskatchewan, Canada: Impact on consumption and implications for public health. American Journal of Public Health, 102, e103-e110. [minimum price elasticities].

**Su, S-J. and Yen, S.T. (2000). A censored system of cigarette and alcohol consumption. Applied Economics, 32, 729-37. [no prices]

**Swidler, S. (1986). Consumption and price effects of state-run liquor monopolies. Managerial and Decision Economics, 7, 49-55. [brand data]

**Tan, A.K.G., Yen, S.T., and Nayga, R.M. (2009). Factors affecting alcohol purchase decisions and expenditures: A sample selection analysis in Malaysia. Journal of Family and Economic Issues, 30, 149-59.[no prices]

**Taylor, L.D. (1968) Personal consumption expenditures in Sweden: 1931-1958. Review of the International Statistical Institute, 36, 19-36. [older data, poor categories]

** Terrien, C. and Steichen, D. (2008). Accounting for social taste: Application to the demand for wine. International Journal of Wine Business Research, 20, 260-75. [no elasticities]

**Toma, E.F. (1988). State liquor licensing, implicit contracting, and dry/wet counties. Economic Inquiry, 26, 507-24. [no elasticities]

**Tremblay, C.H. and Tremblay, V.J. (1995). Advertising, price, and welfare: Evidence from the U.S. Brewing industry. Southern Economic Journal, 62, 367-81. [firm data]

**Treno, A.J. et al. (1993). Understanding U.S. alcohol consumption with social and economic factors: A multivariate time series analysis, 1950-1986, Journal of Studies on Alcohol, 54, 146-56. [ARMA model]

**Van Der Spuy, T. (2011). Advertising Effectiveness in the Alcoholic Beverage Industry of South Africa, MBA Thesis, Stellenbosch University. [brand data]

**Yen, S.T. (1995). Alternative transformations in a class of limited dependent variable models: Alcohol consumption by US women. Applied Economics Letters, 2, 258-62. [no prices]

**Yen, S.T. (2005). A multivariate sample-selection model: Estimating cigarette and alcohol demands with zero observations. American Journal of Agricultural Economics, 87, 453-66. [no prices]

**Yen, S.T., Yuan, Y., and Liu, X. (2009). Alcohol consumption by men in China: A non-Gaussian censored\ system approach. China Economic Review, 20, 162-73. [no prices]

**Yuan, Y. and Yen, S.T. (2012) Alcohol consumption by individuals in the United States: A sample selection approach. Applied Economics Letters, 19, 1353-58. [no prices]

**Wall, M. and Casswell, S. (2012). Affordability of alcohol as a key driver of alcohol demand in New Zealand: a co-integration analysis, Addiction, doi:10.1111/j.1360-0443.2012.03992x [no std errors]

**Waters, T.M. and Sloan, F.A. (1995). Why do people drink? Tests of the rational addiction model. Applied Economics, 27, 727-36. [no elasticities]

**Wilcox, G.B. (2006). What’s changed? Does beer advertising affect consumption in the United States, international Journal of Advertising, 25, 35-50. [no elasticities]

**Wilcox, G.B. and Kim, K.O.K. (2012). Liquor advertising and consumption in the United States, 1971-2008. International Journal of Advertising, 31, 819-34 [no elasticities, tax rates]

**Zardkoohi, A. and Sheer, A. (1984). Public versus private liquor retailing: An investigation into the behavior of the state governments. Southern Economic Journal, 50, 1058-76. [no elasticity values]

**Zimmerman, P.R. and Benson, B.L. (2007). Alcohol and rape: An “economics-of-crime” perspective, International Review of Law and Economics, 27, 442-73. [tax variables]

**IIA. ADULT DRINKING -- SURVEY STUDIES**

1. *An, R., & Sturm, R. (2011). Does the response to alcohol taxes differ across racial/ethnic groups? Some evidence from 1984-2009 Behavioral Risk Factor Surveillance System. Journal of Mental Health Policy and Economics, 14, 13-23.

2. *Auld, M.C. (2005). Smoking, drinking, and income. Journal of Human Resources, 40, 505-18.

3. *Ayyagari, P., Deb, P., Fletcher, J., Gallo, W., & Sindelar, J.L. (2011). Understanding heterogeneity in price elasticities in the demand for alcohol for older individuals*.* Health Economics. Early view at Wiley Online Library, DOI: 10.1002/hec.1817.

4. *Byrnes, J., Shakeshaft, A., Petrie, D., & Doran, C. (2012). Can harms associated with high-intensity drinking be reduced by increasing the price of alcohol? Drug and Alcohol Review. Early view at Wiley Online Library, doi: 10.1111/j.1465-3362.2012.00482.x.

5. *Dave, D., & Saffer, H. (2008). Alcohol demand and risk preference. Journal of Economic Psychology, 29, 810-31.

6. *Dee, T.S. (1999b). Taxes, alcohol use and traffic fatalities. Unpublished working paper, Swarthmore College. Retrieved at http://www.swarthmore.edu/Documents/academics/economics/Dee.

7. *Farrell, S., Manning, W.G., & Finch, M.D. (2003). Alcohol dependence and the price of alcoholic beverages. Journal of Health Economics, 22, 117-47.

8. *Gius, M. (2002). The effect of taxes on alcohol consumption: An individual level analysis with a correction for aggregate public policy variables. Pennsylvania Economic Review, 11, 76-93.

9. *Hamilton, V., & Hamilton, B.H. (1997). Alcohol and earnings: Does drinking yield a wage premium? Canadian Journal of Economics, 30, 135-51.

10. *Harris, M.N., Ramful, P., & Shao, Z. (2006). An ordered generalized extreme value model with application to alcohol consumption in Australia. Journal of Health Economics, 25, 782-801.

11. *Heeb, J.L., Gmel, G., Zurbrugg, C., Kuo, M., & Rehm, J. (2003). Changes in alcohol consumption following a reduction in the price of spirits: A natural experiment in Switzerland. Addiction, 98, 1433-46.

12. *Kenkel, D.S. (1996). New estimates of the optimal tax for alcohol. Economic Inquiry, 34, 296-319.

13. *Manning, W.G., Blumberg, L., & Moulton, L.H. (1995). The demand for alcohol: The differential response to price. Journal of Health Economics, 14, 123-48.

14. *McLellan, D.L. (2011). Intended and unintended consequences: Effect of state cigarette price on smoking and current, binge, and heavy drinking by demographic group*.* Unpublished Ph.D. dissertation, Heller School for Social Policy and Management, Brandeis University.

15. *Nelson, J.P. (2008). How similar are youth and adult alcohol behaviors? Panel results for excise taxes and outlet density. Atlantic Economic Journal, 36, 89-104.

16. *Rhoads, J.K. (2010). Consequences of tobacco control policies: Intended and unintended. Unpublished Ph.D. dissertation, Department of Economics, University of Illinois at Chicago.

17. *Shi, Y. (2011). Three essays on economics of health behavior in China. Unpublished Ph.D. dissertation, Pardee RAND Graduate School, RAND Corporation.

18. *Sloan, F.A., Reilly, B.A. Reilly, and Schenzler, C. (1995). Effects of tort liability and insurance on heavy drinking and drinking and driving. Journal of Law and Economics, 38, 49-77.

19. *Stout, E.M., Sloan, F.A., Liang, L., & Davies, H.H. (2000). Reducing harmful alcohol-related behaviors: Effective regulatory methods. Journal of Studies on Alcohol, 61, 402-12.

**IIB. EXCLUDED STUDIES – ADULTS**

**Anderson, B. and Moro, M. (2008). Depression Economics and alcohol consumption, Working paper, University College, Dublin. [no prices]

**Anderson, D.M. and Rees, D.I. (2011). Medical marijuana laws, traffic fatalities, and alcohol consumption, Discussion paper No. 6112, IZA, Bonn/ [beer tax results not reported]

**Arcidiacono, P., Sieg, H., and Sloan, F. (2007). Living rationally under the volcano? An empirical analysis of heavy drinking and smoking, International Economic Review, 48, 37-65. [prices not reported]

**Berggren, F. and Sutton, M. (1999). Are frequency and intensity of participation decision-bearing aspects of consumption? An analysis of drinking behaviour, Applied Economics, 31, 865-74. [no prices]

Cutler, D.M. and Glaeser, E. (2005). What explains differences in smoking, drinking, and other health-related behaviors? American Economic Review, 95, 238-42. [no prices]

**Dee, T.S. (2001). Alcohol abuse and economic conditions: Evidence from repeated cross-sections of individual-level data, Health Economics, 10, 257-70. [no tax results]

**Dong, Y. (2009). Microeconometric models with endogeneity – Theoretical and empirical studies, Unpublished Ph.D. dissertation, Boston College. [no prices]

**French, M.T. and Zarkin, G.A. (1995). Is moderate alcohol use related to wages? Evidence from four worksites, Journal of Health Economics, 14, 319-44. [no demand]

**French, M.T. et al. (2006). Price elasticity of demand for malt liquor beer: Findings from a US pilot study, Social Science & Medicine, 62, 2101-111. [experimental study]

**Gustafsson, N-K. (2010). Changes in alcohol availability, price and alcohol-related problems and collectivity of drinking cultures: What happened in Southern and Northern Sweden? Alcohol and Alcoholism, 45, 456-67. [no prices]

**Heien, D. (1996). The relationship between alcohol consumption and earnings, Journal of Studies on Alcohol, 57, 536-42. [no demand]

**Heien, D. (1996). Do drinkers earn less? Southern Economic Journal, 63, 60-8. [no demand]

**Ippolito, R.A. (2003). The health effects of alcohol: Do controls for demographics and other risky habits affect the conclusions: Law and Economics Working Paper, George Mason University School of Law [no prices]

**Jimenez, S. and Labeaga, J.M. (1994). Is it possible to reduce tobacco consumption via alcohol taxation, Health Economics, 3, 231-41. [no results for alcohol alone]

**Kerr, W.C. et al. (2012), Are the 1976-1985 birth cohorts heavier drinkers? Age-period-cohort analyses of the National Alcohol Surveys 1979-2010, Addiction (postprint) doi: 10.1111/j.1360-0443.2012.04055.x [no prices]

**Kerr, W.C. et al. (2004). Age, period and cohort influences on beer, wine and spirits consumption trends in the US National Alcohol Surveys, Addiction, 99, 1111-120. [no prices]

**Kuo, M., et al. (2003). Does price matter? The effect of decreased price on spirits consumption in Switzerland, Alcoholism: Clinical and Experimental Research, 27, 720-25. [duplicate study]

**Lye, J.N. and Hirschberg, J. (2004). Alcohol consumption, smoking and wages, Applied Economics, 36, 1807-17. [no demand]

**Nayga, R.M. and Capps, O. (1994). Analysis of alcohol consumption in the United States: Probability and level of intake. Journal of Food Distribution Research, 25, 17-23. [income only, no prices, survey data]

**Ohsfeldt, R.L. and Morrisey, M.A., Beer taxes, workers’ compensation, and industrial injury, Review of Economics and Statistics, 79, 155-59. [no demand]

**Pompelli, G. and Heien, D. (1991). Discrete/continuous consumer demand choices: An application to the U.S. domestic and imported white wine markets, European Review of Agricultural Economics, 18, 117-30. [heavy-light distinction not relevant]

**Preckel, P.V. et al. (2010). A modified, implicit, directly additive demand system, Applied Economics, 42, 143-55. [no separate results for alcohol]

**Rice, N. and Sutton, M. (1998). Drinking patterns within households: The estimation and interpretation of individual and group variables. Health Economics, 7, 689-99. [no prices]

**Ruhm, C.J. and Black, W.E. (2002). Does drinking really decrease in bad times? Journal of Health Economics, 21, 659-78. [no prices]

Saffer, H. and Dave, D. (2005), The effect of alcohol consumption on the earnings of older workers, in Substance Use: Individual Behaviour, Social Interactions, Markets and Politics. Advances in Health Economics and Health Services Recearch, 16, 61-90. [no demand]

**Terza, J.V. (2002). Alcohol abuse and employment: A second look, Journal of Applied Econometrics, 17, 393-404. [no demand]

**Thies, C.F. and Register, C.A. (1993). Decriminalization of marijuana and the demand for alcohol, marijuana and cocaine, Social Science Journal, 30, 385-99. [no prices]

**Van Hoa, T. (1968). Interregional elasticities and aggregation bias: A study of consumer demand in Australia, Australian Economic Paper, 7, 206-26. [no separate results for alcohol]

**Yakovlev, E. (2011). Peers and alcohol: Evidence from Russia, Working paper, University of California, Berkeley. [no std errors]

**IIC. LIVER CIRRHOSIS MORTALITY STUDIES**

1. *Bielinska-Kwapisz, A., & Mielecka-Kubien, Z. (2011). Alcohol consumption and its adverse effect in Poland in year 1950-2005*.* Economics Research International 2011: Article ID 870714, 1-13. doi:10.1155/2011/870714

2. *Cook, P.J., & Tauchen, G. (1982). The effect of liquor taxes on heavy drinking. Bell Journal of Economics, 13, 379-89.

3. *Grossman, M. (1993). The economic analysis of addictive behavior. In M.E. Hilton and G. Bloss (Eds.), Economics and the prevention of alcohol-related problems, Research monograph No. 25 (pp. 91-124). Washington, DC: National Institutes of Health.

4. *Heien, D., & Pompelli, G. (1987). Stress, ethnic and distribution factors in a dichotomous response model of alcohol abuse. Journal of Studies on Alcohol, 48, 450-55.

5. *Nelson, J.P., & Young, D.J. (2001). Do advertising bans work? An international comparison. *International* Journal of Advertising, 20, 273-96.

6. *Ponicki, W.R., & Gruenewald, P.J. (2006). The impact of alcohol taxation on liver cirrhosis mortality. Journal of Studies on Alcohol, 67, 934-38.

7. *Saffer, H. (1991). Alcohol advertising bans and alcohol abuse: An international comparison. Journal of Health Economics, 10*,* 65-79.

8. *Sloan, F.A., Reilly, B.A., & Schenzler, C. (1994). Effects of prices, civil and criminal sanctions, and law enforcement on alcohol-related mortality. Journal of Studies on Alcohol, 55, 454-65.

9. *Wagenaar, A.C., Maldonado-Molina, M.M., & Wagenaar, B.H. (2009a). Effect of alcohol tax increases on alcohol-related disease mortality in Alaska: Time-series analyses from 1976 to 2004. American Journal of Public Health, 99, 1464-70.

**IID. EXCLUDED STUDIES – CIRRHOSIS & MORTALITY**

**Bentzen, J. and Smith, V. (2011). Alcohol consumption and liver cirrhosis mortality: New evidence from a panel data analysis for sixteen European countries, Journal of Wine Economics, 1, 67-82. [no taxes]

**Bhattacharya, J., Gathmann, C., and Miller, G. (2011). The Gorbachev anti-alcohol campaign and Russia’s mortality crisis, Working paper, University of Mannheim. [no prices]

**Bloomfield, K. et al. (2010). Changes in alcohol-related problems after alcohol policy changes in Denmark, Finland, and Sweden, Journal of Studies on Alcohol and Drugs, 71, 32-40. [dependency]

**Brainerd, E. and Varavikova, E.A. (2006). Death and the market, Working paper, Williams College. [total mortality, no prices]

**Brown, R.W. and Jewell, R.T. (1996). County-level alcohol availability and cirrhosis mortality, Eastern Economic Journal, 22, 291-301. [no prices]

**Carpenter, C. and Dobkin, C. (2009). The effect of alcohol consumption on mortality: Regression discontinuity evidence from the minimum drinking age, American Economic Journal: Applied Economics, 1, 164-82. [no prices]

**Di Castelnuovo, A. et al. (2006). Alcohol dosing and total mortality in men and women: An updated meta-analysis of 34 prospective studies, Archives of Internal Medicine, 166, 2437-45. [no prices]

**Cook, P. (1984). Increasing the federal alcohol excise tax, in D.R. Gerstein (ed.), Toward Prevention of Alcohol Problems: Government, Business, and Community Action. Washington, DC: National Academy Press (pp. 24-38). [literature review]

**Cook, P.J. (1981). The effect of liquor taxes on drinking, cirrhosis, and auto accidents, in M.H. Moore and D.R. Gerstein (eds.), Alcohol and Public Policy: Beyond the Shadow of Prohibition. Washington, DC: National Academy Press (pp. 255-85). [duplicate study]

**Cook, P.J. (1987). The impact of distilled-spirits taxes on consumption, auto fatalities, and cirrhosis mortality, in Control Issues in Alcohol Abuse Prevention: Strategies for States and Communities. Advances in Substance Abuse, Suppl. 1, 159-67. [duplicate study]

**Cook, P., Ostermann, J., and Sloan, F.A. (2005). Are alcohol excise taxes good for us? Short and long-term effects on mortality rates, NBER Working Paper 11138. Cambridge: NBER. [total mortality]

**Cook, P.J., Ostermann, J., and Sloan, F.A. (2005), The net effect of an alcohol tax increase on death rates in middle age, American Economic Review, 95, 278-81. [total mortality]

**Dills, A.K. and Miron, J.A. (2004). Alcohol prohibition and cirrhosis, American Law and Economics Review, 6, 285-318. [older data]

**Fuller, T.D. (2011). Moderate alcohol consumption and risk of mortality, Demography, 48, 1105-25. [total mortality]

Gmel, G. et al. (2001). Methodological approaches to conducting pooled cross-sectional time series analysis: The example of the association between all-cause mortality and per capital alcohol consumption for men in 15 European states, European Addiction Research, 7, 128-37. [total mortality]

Gronbaek, M. (2001). Factors influencing the relation between alcohol and mortality – with focus on wine, Journal of Internal Medicine, 250, 291-308. [literature review]

**Gruenewald, P.J. and Ponicki, W.R. (1995). The relationship of alcohol sales to cirrhosis mortality, Journal of Studies on Alcohol, 56, 635-41. [no prices]

**Harford, T.C. and Brooks, S.D. (1992), Cirrhosis mortality and occupation, Journal of Studies on Alcohol, 53, 463-68. [no prices]

**Henderson, C. et al. (2004). The effects of US state income inequality and alcohol policies on symptoms of depression and alcohol dependence, Social Science & Medicine, 58, 565-75. [dependence]

**Her, M. and Rehm, J. (1998). Alcohol and all-cause mortality in Europe 1982-1990: A pooled cross-section time-series analysis, Addiction, 93, 1335-40. [no prices]

**Herttua, K. et al. (2008). Changes in alcohol-related mortality and its socioeconomic differences after a large reduction in alcohol prices: A natural experiment based on register data, American Journal of Epidemiology, 168, 1110-1118. [total mortality]

**Holahan, C.J. et al. (2010). Late-life alcohol consumption and 20-year mortality, Alcoholism: clinical and Experimental Research, 334, 1961-71. [total mortality]

**Kendell, R.E. et al. (1983). Effect of economic changes on Scottish drinking habits 1978-82, British Journal of Addiction, 78, 365-79. [no regressions]

**Lin, C-M. et al. (2011). A time-series analysis of alcohol tax policy in relation to mortality from alcohol attributed causes in Taiwan, Journal of Community Health, 36, 986-91. [total mortality]

**Maldonado-Molina, M. and Wagenaar, A.C. (2010). Effects of alcohol taxes on alcohol-related mortality in Florida: Time –series analysis from 1969 to 2004, Alcoholism: Clinical and Experimental Research, 34, 1915-21. [total mortality]

**Mullahy, J. and Sindelar, J.L. (1994). Alcoholism and income: The role of indirect effects, Milbank Quarterly, 72, 359-75. [no prices]

**Mullahy, J. and Sincelar, J. (1989). Life-cycle effects of alcoholism on education, earning, and occupation, Inquiry, 26, 272-82. [no prices]

**Nielsen, N.R. et al. (2004). Is the relationship between type of alcohol and mortality influenced by socio-economic status? Journal of Internal Medicine, 255, 280-88. [no prices]

**Petrie, D. et al. (2007). The relationship between alcohol consumption and self-reported health status using the EQ5D, Working paper, University of Dundee [no prices]

**Rehm, J. et al. (2006). Patterns of drinking and mortality from different diseases – an overview, Contemporary Drug Problems, 33, 205-35. [literature review]

Rehm, J. and Sempos, C.T. (1995). Alcohol consumption and all-cause mortality, Addiction, 471-80. [total mortality]

**Roizen, R. et al. (1999). Cirrhosis mortality and per capita consumption of distilled spirits, United States, 1949-94: Trend analysis, BM, 219, 666-70. [no regression]

**Ruhm, C.J. (2000). Are recessions good for your health? Quarterly Journal of Economics, 115, 617-50. [no prices]

**Ruhm, C.J. and Gerdtham, U-G. (2006). Deaths rise in good economic times: Evidence from the OECD, Economics and Human Biology, 4, 298-316. [no prices]

**Rush, B. et al. (1986). The relationship among alcohol availability, alcohol consumption and alcohol-related damage in the province of Ontario and the state of Michigan, Advances in Alcohol & Substance Abuse, 5, 33-45. [simple correlation model]

**Skog, O-J. (2003). Alcohol consumption and fatal accidents in Canada, 1950-98, Addiction, 98, 883-93. [accidents]

**Smart, R.G. and Mann, R.E. (1998). Treatment, alcoholics anonymous and alcohol controls during the decrease in alcohol problems in Alberta, 1975-1993, Alcohol & Alcoholism, 33, 265-72. [simple correlations model]

**Son, C.H. and Topyan, K. (2011). European Journal of Health Economics, 12, 103-113. [accidents]

**Thun, M.J. et al. (1997). Alcohol consumption and mortality among middle-aged and elderly U.S. adults, New England Journal of Medicine, 337, 1705-14. [total mortality]

**Walsh, B.M. (1987). Do excise taxes save lives? The Irish experience with alcohol taxation, Accident Analysis & Prevention, 19, 433-48. [before-after study, no taxes in regression]

**Wilson, R.A. (1984). Changing validity of the cirrhosis mortality-alcoholic beverage sales construct: U.S. trends, 1970-1977, Journal of Studies on Alcohol, 45, 53-8. [no prices]

**Young, D.J. (1993). Alcohol advertising bans and alcohol abuse: comment, Journal of Health Economics, 12, 213-28. [duplicate study]

**IIIA. GENDER-RELATED DRINKING SURVEY STUDIES: ADULTS**

1. *Andrienko, Y. and A. Nemtsov (2006). Estimation of individual demand for alcohol. Unpublished paper, Centre for Economic and Financial Research, New Economic School.

2. *Auld, M.C. (2005). Smoking, drinking, and income. Journal of Human Resources 40: 505-18.

3. *Baltagi, B.H. and I. Geishecker (2006). Rational alcohol addiction: Evidence from the Russian longitudinal monitoring survey. Health Economics 15: 893-914.

4. *Belanciuc, A. (2006). Estimation of individual demand for alcohol: Evidence from Ukraine. Unpublished Masters Thesis, National University of Kyiv-Mohyla Academy.

5. *Decker, S.L. and A.E. Schwartz (2000). Cigarettes and alcohol: Substitutes or complements. NBER Working Paper No. 7535. Cambridge, MA.

6. *Dee, T.S. (1999b). Taxes, alcohol use and traffic fatalities. Unpublished working paper, Swarthmore College.

7. *Hamilton, V. and B.H. Hamilton (1997). Alcohol and earnings: Does drinking yield a wage premium? Canadian Journal of Economics 30: 135-51.

8. *Kenkel, D.S. (1993). Drinking, driving, and deterrence: The effectiveness and social costs of alternative policies. Journal of Law and Economics 36: 877-913.

9. *Kenkel, D.S. (1996). New estimates of the optimal tax for alcohol. Economic Inquiry 34: 296-319.

10. *Picone, G.A., F. Sloan, and J.G. Trogdon (2004). The effect of tobacco settlement and smoking bans on alcohol consumption. Health Economics 13: 1063-80.

11. *Pierani, P. and S. Tiezzi (2011). Infrequency of purchase, individual heterogeneity and rational addiction in single households’ estimates of alcohol consumption. Giornale degli Economisti e Annali de Economia 70: 93-116.

12. *Shi, Y. (2011). Three essays on economics of health behavior in China. Unpublished Ph.D. dissertation, Pardee RAND Graduate School, RAND Corporation.

13. *Tian, G. and F. Liu (2011). Is the demand for alcoholic beverages in developing countries sensitive to price? Evidence from China. International Journal of Environmental Research and Public Health 8: 2124-32. doi:10.3390/ijerph8062124.

14. *West, S.E. and I.W.H. Parry (2009). Alcohol/leisure complementarity: Empirical estimates and implications for tax policy. National Tax Journal 62: 611-34.

15. *Zhang, N. (2010). Alcohol taxes and birth outcomes. International Journal of Environmental Research and Public Policy 7: 1901-12. doi:10.3390/ijerph7051901.

**IIIB. EXCLUDED STUDIES: GENDER – ADULTS**

**Asgeirsdottir, T.L. and McGeary, K.A. (2009). Alcohol and labor supply: The case of Iceland, European Journal of Health Economics, 10, 455-65. [no prices, no demand]

**Baltagi, B.H. (2007). On the use of panel data methods to estimate rational addiction models, Scottish Journal of Political Economy, 54, 1-18. [literature review]

**Berggren, F. and Sutton, M. (1999). Are frequency and intensity of participation decision-bearing aspects of consumption? An analysis of drinking behaviour, Applied Economics, 31, 865-74. [no prices]

**Browning, M. (1987). Eating, drinking, smoking, and testing the lifecycle hypothesis, Quarterly Journal of Economics, 92, 329-45. [no price results by gender]

**Cameron, L. and Williams, J. (2001). Cannabis, alcohol and cigarettes: Substitutes or complements? Economic Record, 77, 19-34. [no gender results]

**Carpenter, C. and Harris, K. (2005). How do “point oh-eight” (.08) BAC laws work? Topics in Economic Analysis and Policy, 5, Article 6. [taxes not reported]

**Chatterji, P. and DeSimone, J. (2006). High school alcohol use and young adult labor market outcomes, NBER Working paper 12529. [no demand]

**Cook, P.A., et al. (2011). The big drink debate: Perceptions of the impact of price on alcohol consumption from a large scale cross-sectional convenience survey in northwest England, BMC Public Health, 11, 664. [no elasticities]

**Cook, P., Ostermann, J., and Sloan, F.A. (2005). Are alcohol excise taxes good for us? Short and long-term effects on mortality rates, NBER Working Paper 11138. Cambridge: NBER. [no gender results for taxes]

**Dave, D. and Kaestner, R. (2002). Alcohol taxes and labor market outcomes, Journal of Health Economics, 21, 357-71. [no demand]

**Dee, T.S. (2001). Alcohol abuse and economic conditions: Evidence from repeated cross-sections of individual-level data, Health Economics, 10, 257-70. [no tax results]

**Dong, Y. (2009). Microeconometric models with endogeneity – Theoretical and empirical studies, Unpublished Ph.D. dissertation, Boston College. [no prices]

**Eisenberg, D. (2001). Evaluating the effectiveness of a 0.08% BAC limit and other policies related to drunk driving, SIEPR Discussion paper No. 00-23, Stanford University. [no demand]

**Grittner, U. et al. (2009). Changes in alcohol consumption in Denmark after the tax reduction in spirits, European Addiction Research, 15, 216-23. [before-after study, no taxes]

**Gerolimetta, M., Mauracher, C., and Procidano, I. (2008). Analyzing wine demand with artificial neural networks. Journal of Wine Economics, 3, 30-50. [no std. errors]

**Gustafsson, N-K. (2010). Changes in alcohol availability, price and alcohol-related problems and collectivity of drinking cultures: What happened in Southern and Northern Sweden? Alcohol and Alcoholism, 45, 456-67. [no prices]

**Harris, M.N., Ramful, P., & Shao, Z. (2006). An ordered generalized extreme value model with application to alcohol consumption in Australia. Journal of Health Economics, 25, 782-801. [gender results only summarized]

**Heeb, J-L, et al. (2003). Changes in alcohol consumption following a reduction in the price of spirits: A natural experiment in Switzerland, Addiction, 98, 1433-46. [experimental study]

**Kaestner, R. and Yarnoff, B. (2011). Long-term effects of minimum legal drinking age laws on adult alcohol use and driving fatalities, Journal of Law and Economics, 54, 365-88. [taxes not reported]

**Kuo, M., et al. (2003). Does price matter? The effect of decreased price on spirits consumption in Switzerland, Alcoholism: Clinical and Experimental Research, 27, 720-25. [experimental study]

**Madden, D. (2008). Sample selection versus two-part models revisited: The case of female smoking and drinking. Journal of Health Economics, 27, 300-07. [no prices]

**Mullahy, J. and Sindelar, J. (1996). Employment, unemployment, and problem drinking, Journal of Health Economics, 15, 409-34. [no demand]

**Mullahy, J. and Sindelar, J.L. (1994). Do drinkers know when to say when? An empirical analysis of drunk driving, Economic Inquiry, 32, 383-94. [no demand]

**Mullahy, J. and Sindelar, J.L. (1998). Drinking, problem drinking, and productivity, Recent Developments in Alcoholism, 14, 347-59. [literature review] 10.

*Peters, B.L. (2004). Is there a wage bonus for drinking? Unobserved heterogeneity examined, Applied Economics, 36, 2299-315. [no demand estimates]

**Peters, B.L. and Stringham, E. (2006). No booze? You lose: Why drinkers earn more money than nondrinkers, Journal of Labor Research, 27, 411-21. [no prices]

**Petrie, D., et al. (2009). The demand for intensity versus frequency of alcohol consumption: Evidence from rural Australia, Working paper No. 222, University of Dundee. [no prices]

**Rice, N. et al. (1998). The influence of households on drinking behaviour: A multilevel analysis, Social Science & Medicine, 46, 971-79. [no prices]

**Saffer, H. et al. (2012). Behavioral economics and the demand for alcohol: results from the NLSY97, NBER Working paper 18180. [no gender results]

**Srivastava, P. (2010). Does bingeing affect earnings? Economic Record, 86, 578-95. [no demand]

**Tauchmann, et al. (2007). Tobacco and alcohol: Complements or substitutes – A structural model approach, Ruhr Economic Papers No. 34, University of Kiel. [no prices]

**Terza, J.V. et al. (2008). Care-giver advice as a preventive measure for drinking during pregnancy: Zeros, categorical outcome responses, and endogeneity, Health Economics, 17, 41-54. [demand not reported]

**Yen, S.T. (2005). A multivariate sample-selection model: Estimating cigarette and alcohol demands with zero observations. American Journal of Agricultural Economics, 87, 453-66. [no prices]

**Yen, S.T. (1995). Alternative transformations in a class of limited dependent variable models: Alcohol consumption by US women. Applied Economics Letters, 2, 258-62. [no prices]

**Yen, S.T., Yuan, Y., and Liu, X. (2009). Alcohol consumption by men in China: A non-Gaussian censored\ system approach. China Economic Review, 20, 162-73. [no prices]

**Yuan, Y. and Yen, S.T. (2012) Alcohol consumption by individuals in the United States: A sample selection approach. Applied Economics Letters, 19, 1353-58. [no prices]

**Zarkin, G.A. et al. (1998). Alcohol use and wages: New results from the National Household Survey on Drug Abuse, Journal of Health Economics, 17, 53-68. [no demand]

**Ziebarth, N.R. and Grabka, M.M. (2009). In vino pecunia? The association between beverage-specific drinking behavior and wages, Journal of Labor Research, 30, 219-34. [no demand]

**Zimmerman, P.R. and Benson, B.L. (2007). Alcohol and rape: An “economics-of-crime” perspective, International Review of Law and Economics, 27, 442-73. [no gender results for demands]

**IIIC. GENDER-RELATED DRINKING SURVEY STUDIES: YOUNG ADULTS (8 studies)**

1. *Chaloupka, F.J. and H. Wechsler (1996). Binge drinking in college: The impact of price, availability and alcohol control problems. Contemporary Economic Problems 14: 112-24.

2. *Cook, P.J. and M.J. Moore (2001). Environment and persistence in youthful drinking. In: J. Gruber (ed.), Risky behavior among youths: An economic analysis (pp. 375-437). Chicago: University of Chicago Press.

3. *French, M.T. and J.C. Maclean (2006). Underage alcohol use, delinquency, and criminal activity. Health Economics 15: 1261-81.

4. *Keng, S-H. (1998). The demand for health, alcohol abuse, and labor market outcomes: A longitudinal study. Unpublished Ph.D. dissertation, Department of Economics, Iowa State University.

5. *Kenkel, D.S. (1993). Drinking, driving, and deterrence: The effectiveness and social costs of alternative policies. Journal of Law and Economics 36: 877-913.

6. *Shi, Y. (2011). Three essays on economics of health behavior in China. Unpublished Ph.D. dissertation, Pardee RAND Graduate School, RAND Corporation.

7. *Sutton, M. and C. Godfrey (1995). A grouped data regression approach to estimating economic and social influences on individual drinking behaviour. Health Economics 4: 237-47.

8. *Williams, J., F.J. Chaloupka, and H. Wechsler (2002). Are there differential effects of price and policy on college students drinking intensity? National Bureau of Economic Research Working Paper 8702. Cambridge, MA.

**IIID. EXCLUDED STUDIES: GENDER – YOUNG ADULTS**

**Argys, L.M. et al. (2006). Birth order and ricky adolescent behavior, Economic Inquiry, 44, 215-33. [ages 12-17 years]

**Bellis, M.A. et al. (2009). Teenage drinking, alcohol availability and pricing: A cross-sectional study of risk and protective factors for alcohol-related harms in school children, BMC Public Health, 9, 380. [ages 15-16 years]

**Bhatt, V. (2011). Adolescent alcohol use and intergenerational transfers: Evidence from micro data, Journal of Family Economic Issues, 32, 296-307. [ages 12-18 years]

**Bishai, D.M. et al. (2005). Can government policies help adolescents avoid risky behavior, Preventive Medicine, 40, 197-202. [mean age 16 years, no std errors]

**Bryant, R.R. and Samaranayake, V.A. (1992). Alcohol use and wages of young men: Whites vs nonwhites, International Review of Applied Economics, 6, 184-202. [no demand]

**Carpenter, C. (2004). How do zero tolerance drunk driving laws work? Journal of Health Economics, 23, 61-83. [no prices]

**Carpenter, C. (2004). Heavy alcohol use and youth suicide: Evidence from tougher drunk driving laws, Journal of Policy Analysis and Management, 23, 831-42. [no demand]

**Chaloupka, F.J. and Laixuthai, A. (1997). Do youths substitute alcohol and marijuana? Some econometric evidence, Eastern Economic Journal, 23, 253-76. [MTF data]

**Chatterji, P. (2001). What determines adolescent demand for alcohol and marijuana? A comparison of the findings from the NLSY79 and NLSY97, in R.T. Michael (ed.), Social Awakening: Adolescent Behavior as Adulthood Approaches. New York: Russell Sage, pp. 299-38. [adolescents]

**Cook, M.J. (2008). Underage alcohol consumption in the United States: Associations with access laws, alcohol excise taxes and enforcement practices, Unpublished Ph.D. dissertation, University of Connecticut. [no regressions]

**Cook, P.J. and Moore, M.J. (1994). This tax’s for you: the case for higher beer taxes. National Tax Journal 47, 559-73. [earlier version of 2001 study, duplicate]

**Dee, T.S. (2000). The effect of alcohol use and availability on teen childbearing, Working paper, Swarthmore College. [NELS data]

**Dee, T.S. and Evans, W.N. (2003). Teen drinking and educational attainment: Evidence from two-sample instrumental variable estimates, Journal of Labor Economics, 21, 178-209. [NELS data]

**Delaney, L. et al. (2006). Behavioural economics and drinking behaviour: Preliminary results from an Irish college study, Working paper, University College Dublin. [no prices]

DeSimone, J. (2007). Fraternity membership and binge drinking, Journal of Health Economics, 26, 950-67 [no prices]

DeSimone, J. (2009). Fraternity membership and drinking behavior, Economic Inquiry, 47, 337-50. [no prices].

**DiNardo, J. and Lemieux, T. (2001). Alcohol, marijuana, and American youth: The unintended consequences of government regulation, Journal of Health Economics, 20, 991-1010. [MTF data]

**Fletcher, J.M. (2012). Peer influences on adolescent alcohol consumption: Evidence using an instrumental variables/fixed effect approach, Journal of Population Economics, 25, 1265-86. [no prices]

**Freeman, D.G. (2011). Is beer healthier than booze? How the change in consumption shares of alcoholic beverage types affects mortality in young people, SHSU Working paper, Sam Houston State University [no prices]

**Gius, M.P., An estimate of the effects of age, taxes, and other socioeconomic variables on the alcoholic beverage demand of young adults, Social Science Journal, 42, 13-24. [no gender results]

**Gius, M.P. (2003). Using NLSY-Geocode data to determine the effects of taxes and minimum age laws on the alcoholic beverage demand of young adults, New York Economic Review, 34, 38-50. [no gender results]

Grossman, M. and Markowitz, S. (1999). Alcohol regulation and violence on college campuses, in M. Grossman and C-R. Hsieh (eds.), Economic Analysis of Substance Use and Abuse. Cheltenham: Elgar, pp. 257-89. [no gender results]

**Heeb, J-L, et al. (2003). Changes in alcohol consumption following a reduction in the price of spirits: A natural experiment in Switzerland, Addiction, 98, 1433-46. [experimental study]

**Kaestner, R. (2000). A note on the effect of minimum drinking age laws on youth alcohol consumption, Contemporary Economic Problems, 18, 315-25. [no prices]

**Keng, S-H. and Huffman, W.E. (2007). Binge drinking and labor market success: A longitudinal study on young people, Journal of Population Economics, 20, 35-54. [no gender results]

**Kenkel, D.S. and Ribar, D.C. (1994). Alcohol consumption and young adults’ socioeconomic status, Brookings Papers: Microeconomics, 1994, 119-75. [no prices]

**Koch, S.F. and Ribar, D.C. (2001). A siblings analysis of the effects of alcohol consumption onset on educational attainment, Contemporary Economic Policy, 19, 162-74. [no prices]

**Kuo, M. et al. (2003). The marketing of alcohol to college students: The role of low prices and special promotions, American Journal of Preventive Medicine, 25, 204-11. [no prices]

**Kremer, M. and Levy, D. (2008). Peer effects and alcohol use among college students, Journal of Economic Perspectives, 22, 189-206. [no prices]

**Laixuthai, A. and Chaloupka, F.J. (1993). Youth alcohol use and public policy, Contemporary Economic Problems, 11, 70-81. [MTF data]

**Lee, Y.G. and Abdel-Ghany, M. (2004). American youth consumption of licit and illicit substances, International Journal of Consumer Studies, 28, 454-65. [no prices]

**Lundborg, P. (2002). Young people and alcohol: An econometric analysis, Addiction, 97, 1573-82. [ages 12-18 years]

**Lundborg, P. and Lindgren, B. (2002). Risk perceptions an and alcohol consumption, Journal of Risk and Uncertainty, 25, 165-83. [ages 12-18 years]

**Markowitz, S. and Tauras, J. (2009). Substance use among adolescent students with consideration of budget constraints, Review of Economics of the Household, 7, 423-46. [ages 12-16 years]

**McLellan, D.L. (2011). Intended and unintended consequences: Effects of state cigarette price on smoking and current, binge, and heavy drinking by demographic group, Unpublished Ph.D. dissertation, Brandeis University. [no separate gender results for alcohol]

**McLellan, D.L. (2012). Unintended consequences of cigarette price changes for alcohol drinking behaviors across age groups: Evidence from pooled cross sections, Substance Abuse Treatment, Prevention, and Policy, 7, 28. [price results not reported]

**Medina, J.L. (2011). Smoking, drinking, and binge drinking: An empirical study of the role of price on consumption by high school seniors, Unpublished Ph.D. dissertation, City University of New York. [high school students]

**Miller, T. et al. (2006). Retail alcohol monopolies, underage drinking, and youth impaired driving deaths, Accident Analysis and Prevention, 38, 1162-67. [no prices]

**Murry, J.P. et al. (1993). Evaluating an anti-drinking and driving advertising campaign with a sample survey and time series intervention analysis, Journal of the American Statistical Association, 88, 50-56. [no prices]

**Nair, R. et al. (1999). Gender and race differences in youth alcohol demand, in M. Grossman and C-R. Hsieh (eds.), Economic Analysis of Substance Use and Abuse. Cheltenham: Elgar, pp. 391-411. [MTF data]

**Nelson, T.F., et al. (2005). The state sets the rate: The relationship among state-specific college, binge drinking, state binge drinking rates, and selected state alcohol control policies, American Journal of Public Health, 95, 441-46. [no prices]

**Norton, E.C. et al. (1998). Controlling for the endogeneity of peer substance use on adolescent alcohol and tobacco use, Health Economics, 7, 439-53. [upper elementary school students]

**O’Mara, R.J. et al. (2009). Alcohol price and intoxication in college bars, Alcoholism: Clinical and Experimental Research, 33, 1973-80. [experimental study]

**Paschall, M.J. et al. (2009). Alcohol control policies and alcohol consumption by youth: A multi-national study, Addiction 104, 1849-55. [ages 15-17 years]

**Powell, L.M. et al. (2002). Study habits and level of alcohol use among college students, Research Paper No. 19, ImpacTeen. [no gender results, no prices]

**Powell, L.M. et al. (2002). Binge drinking and violence among college students: Sensitivity to correlation in the unobservables, Research Paper No. 20, ImpacTeen. [no gender results, no prices]

**Rashad, I. and Kaestner, R. (2004). Teenage sex, drugs and alcohol use: Problems identifying the cause of risky behaviors, Journal of Health Economics, 23, 493-503. [no demand]

**Renna, F. (2007). The economic cost of teen drinking: Late graduation and lowered earnings, Health Economics, 16, 407-19. [age 18 years]

**Saffer, H. and Chaloupka, F.J. (1999), Demographic differential in the demand for alcohol and illicit drugs, in in M. Grossman and C-R. Hsieh (eds.), Economic Analysis of Substance Use and Abuse. Cheltenham: Elgar, pp. 187-211. [women, but no separate age results]

**Sander, W. (1999). Cognitive ability, schooling and the demand for alcohol by young adults, Education Economics, 7, 53-66. [no prices]

**Schmidt, C.M. and Tauchmann, H. (2011). Heterogeneity in the intergenerational transmission of alcohol consumption: A quantile regression approach, Journal of Health Economics, 30, 33-42. [no prices]

**Srivastava, P. and Zhao, X. (2010). What do bingers drink? Micro-unit evidence on negative externalities and drinker characteristics of alcohol consumption by beverage types, Economic Papers, 29, 229-50. [no regressions]

**Wechsler, H. et al. (1995). Correlates of college student binge drinking, American Journal of Public Health, 85, 921-26. [no prices]

**Wechsler, H. et al. (2000). Environmental correlates of underage alcohol use and related problems of college students, American Journal of Preventive Medicine, 19, 24-9. [no prices]

**Wechsler, J. et al. (1997). Binge drinking among college students: A comparison of California with other states, College Health, 45, 273-77. [no prices]

**Willams, J. (2005). Habit formation and college students’ demand for alcohol, Health Economics, 14, 119-34. [no gender results]

**Williams, J. et al. (2004). Alcohol and marijuana use among college students: Economic complements or substitutes, Health Economics, 13, 825-43. [no prices in gender models]

**Williams, J. et al. (2003). Does alcohol consumption reduce human capital accumulation? Evidence from the College Alcohol Study, Applied Economics, 35, 1227-39. [no gender results]

**Williams, J. et al. (2005). Are there differential effect of price and policies on college students’ drinking intensity, Contemporary Economic Policy, 23, 78-90. [no gender results]

**Wolaver, A.M. (2002). Effects of heavy drinking in college on study effort, grade point average, and major choice, Contemporary Economic Policy, 20, 415-28. [no prices]

**Wolaver, A. et al. (2007). What matters: Reality or perception? The impact of peer binging on college student drinking behaviors, Research Paper No. 36, ImpactTeen. [no gender results]

Zhao, X. and Harris, M.N. (d004). Demand for marijuana, alcohol and tobacco: Participation, levels of consumption and cross-equation correlation, Economic Record, 80, 394-410. [no gender results]

**IV. ADDITIONAL SEARCHES**

In addition, searches were made of the publications, web sites, and vita for several prolific authors, including (among others):

Christopher S. Carpenter

Frank J. Chaloupka

Thomas S. Dee

Donald G. Freeman

Michael Grossman

Christopher J. Ruhm

Saroja Selvanathan

Eliyathamby A. Selvanathan

Kenneth W. Clements

Jenny Williams
